# Mysteries and unsolved problems of mammalian fertilization and related topics

**DOI:** 10.1093/biolre/ioac037

**Published:** 2022-03-15

**Authors:** Ryuzo Yanagimachi

**Affiliations:** Institute for Biogenesis Research, Department of Anatomy, Biochemistry and Physiology, University of Hawaii Medical School, Honolulu, HI 96822, USA

**Keywords:** acrosome, capacitation, egg, fertilization, hyperactivation, oocyte, oviduct, seminal plasma, sperm, spermatozoa

## Abstract

Mammalian fertilization is a fascinating process that leads to the formation of a new individual. Eggs and sperm are complex cells that must meet at the appropriate time and position within the female reproductive tract for successful fertilization. I have been studying various aspects of mammalian fertilization over 60 years. In this review, I discuss many different aspects of mammalian fertilization, some of my laboratory’s contribution to the field, and discuss enigmas and mysteries that remain to be solved.

## Teaser

During the past 60 years, considerable progress has been made in the analysis and manipulation of mammalian gametes and fertilization processes, yet there are many important and interesting topics to be further explored.

## Graphical Abstract

**Graphical Abstract:**
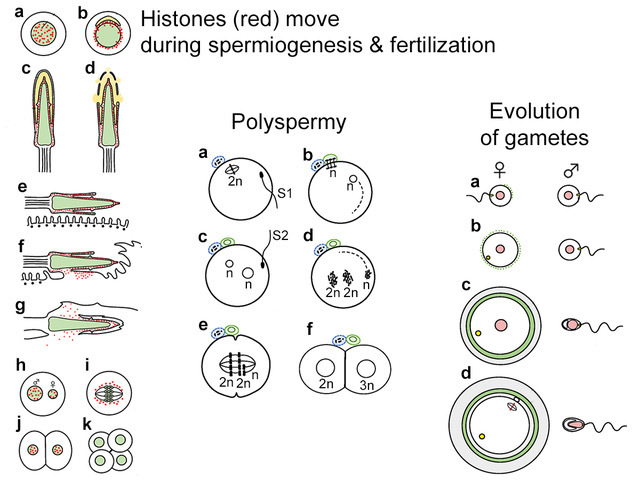

## Contents

1.  Transport of rodent spermatozoa through the vagina, uterus and utero-tubal Junction

2.  Mysteries of the oviduct isthmus

3.  Sperm chemotaxis

4.  Problems of sperm capacitation

5.  Sperm hyperactivation

6.  Time, site, and cause of sperm acrosome reaction *in vivo* and *in vitro*

7.  Oviductin and oviductosome: oviduct's secretory products

8.  Size and shape of spermatozoa

9.  Role of protease/proteasome in sperm capacitation and sperm-egg interaction

10. Role of acrosomal enzymes in fertilization

11. Why is the presence of cumulus oophorus around the egg beneficial for fertilization?

12. How do spermatozoa pass though the zona pellucida of eggs?

13. Is the zona pellucida essential for fertilization and embryo development?

14. The presence of perivitelline space before fertilization: A unique feature in mammals

15. Membrane fusion of sperm and oocyte

16. What is the sperm born oocyte-activating factor?

17. Polyspermy block, with a note on human diploid-triploid mosaics

18. Sperm centrosome and embryo development

19. Fertile life of human oocytes and spermatozoa in oviducts

20. Effect of light on eggs and embryos

21. Puzzles of seminal plasma and sperm competition

22. Similarity between spermatozoa and neurons

23. Intracytoplasmic sperm injection (ICSI): history and challenges to be considered

24. Fertilization by round spermatids and spermatocytes

25. Sperm sexing

26. Conversion of somatic cells to germ cells: artificial gametes

27. Transfer and exchange of sperm chromosomes between two individuals

28. Life without males

29. Human and organ cloning

## Introduction

Analytical study of mammalian fertilization began in the middle of the last century after Austin [1] clarified every step of fertilization by his careful microscopic examination, and Austin [2] and Chang [3] co-discovered that mammalian spermatozoa require capacitation before they become fertilization competent [4]. Thibault et al. [5] first saw a spermatozoon within the egg (of rabbit) after in vitro insemination using capacitated spermatozoa. After that, the use of various species as well as various technologies and approaches, such as electron microscopic, microsurgical, biochemical, molecular approaches, and gene manipulations, greatly enhanced our understanding of the processes of mammalian fertilization. Here, I selected some of the topics that I thought need further research, discussion, and debates.

## 1. Transport of rodent spermatozoa through the vagina, uterus, and utero-tubal junction

It is generally thought that laboratory rodent spermatozoa are inseminated directly into the uterus during coitus. In fact, in the rat and hamster, for example, the bulk of semen (sperm plus seminal plasma) can be recovered from the uterus soon after coitus. Close examination revealed that the semen is deposited deep in the vagina before it is transported to the uterus [6].

Sperm transport from the vagina to the uterus seems to be complex. Here is an example. When hamsters were injected with human chorionic gonadotropin (hCG) 1 day before expected time of luteinizing hormone (LH) release, ovulation occurred about 12 h later as expected, but 15% of the females did not come into behavioral estrus [7]. The remaining 85% of females came into behavioral estrus. When these estrous females were mated and examined 1–2 h later, semen was found in the vagina, but none or only very few spermatozoa were found in the uterus. Apparently, semen transport from the vagina to the uterus does not occur automatically. The release of prolactin from the pituitary triggered by the female’s orgasm [8] may relax the cervix momentarily [9] or induce a “pumping-up” motion of the cervix. Further studies are needed to clarify this point.

When examined soon after mating, the uteri of mated golden hamster females were exhibiting very active ad-oviductal, peristaltic contractions, “pushing” boluses of dense sperm mass to the utero-tubal junctions (UTJs) (Yanagimachi, unpublished observation). It would be interesting to know whether uteri begin active contractions if a sperm suspension in a simple balanced salt solution was introduced into the uterine lumen. If the uteri do not begin active contractions, some components in the seminal plasma or female’s orgasm must play a role in inducing the uterine contractions.

At least in the mouse, a protein on sperm head surface encoded by gene Adam A3 is essential for sperm migration from the uterus into the UTJ [10, 11]. Why Adam A3-null spermatozoa are unable to pass through the UTJ is not completely understood. It is likely that these spermatozoa are unable to attach to the UTJ’s epithelium before swimming through the UTJ. How spermatozoa ascend the UTJ is also mysterious. According to Jungnickel et al. [12], mouse spermatozoa without flagellar protein ENKURIN are unable to exhibit normal flagellar bending. They are far less efficient in entering the oviduct than wild-type spermatozoa. Thus, both motility and surface characteristics of spermatozoa seem to play critical roles in sperm entry from the uterus into the oviduct, at least in the mouse. In the hamster, spermatozoa of the same species migrate from the uterus to the oviduct far more efficiently than those of other species [13]; this migration also requires that spermatozoa be uncapacitated [14].

According to Suarez [15], the lumen of the mouse UTJ is filled with a “mucus.” This mucus and a narrow lumen of the UTJ may prevent spermatozoa from exhibiting large amplitude tail movements. A time-lapse movie (Muro et al. [16]) shows mouse spermatozoa move very slowly toward the isthmus along the smooth inner surface of the UTJ. How such a slow flagellar beat is able to propel spermatozoa forward is another mystery. Qu et al. [17] saw numerous mouse spermatozoa with their heads abutted against the epithelium of the UTJ. They speculated that spermatozoa clustering together in such an orientation and beating their tails synchronously enable the spermatozoa to move into the UTJ. Spermatozoa within the UTJ lumen were no longer clustered. Whether co-operative tail movement by many spermatozoa is really needed for sperm passage through the UTJ must be further investigated. It may be sperm head attachment to the UTJ’s epithelium, not sperm clustering per se, that is of critical importance for sperm entry into the UTJ.

For sperm transport in the female genital tract of other animals, readers are referred to excellent reviews already available [15, 18–20]. We still know very little about sperm transport in human female genital tract. Gene ADAM Metallopeptidase Domain 3 (ADAM3), essential for sperm transport through the female tract of the mouse, is absent in human. Even the longevity (survival) of spermatozoa within women’s genital tract is not certain, with a range of less than 3 days [21] to as long as 25 days [22].

**Figure 1:**
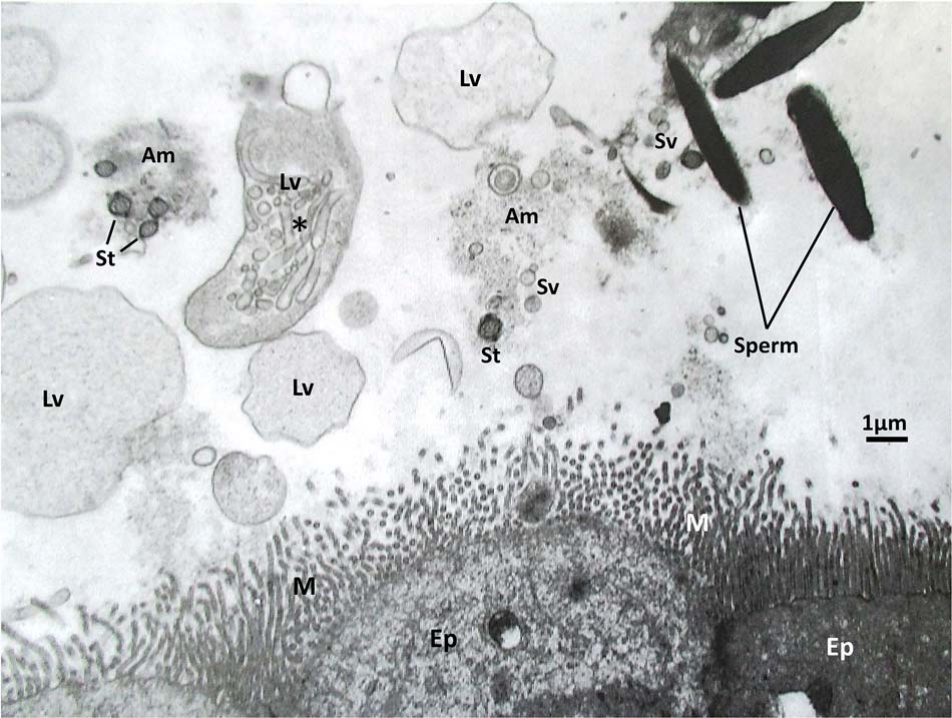
An electron micrograph of mouse spermatozoa in the lumen of oviduct’s isthmus about the time of ovulation after natural mating. Note the presence of many globular and vesicular materials in the isthmus lumen. Am, amorphous material; EP, mucus epithelial cell of isthmus; Lv, large vesicle; M, microvilli of mucus epithelial cell; St, cross section of sperm tail; and Sv, small vesicle. This electron micrograph was prepared by Dr. Kiyotaka Toshimori after perfusion fixation of mouse oviduct.

## 2. Mysteries of the oviduct isthmus

Fertilization is possible without the isthmus of the oviduct. Hunter and Leglise [23] and Patterson et al. [24] surgically removed the entire isthmuses of pig oviducts and connected the ampullas directly to the UTJs. When the pigs were mated, a large proportion of oocytes were fertilized and developed into normal fetuses. Nevertheless, Hunter and Leglise noted that many fertilized eggs were polyspermic, suggesting that the isthmus prevents the migration of excess spermatozoa to the ampulla where fertilization takes place. The functions of the isthmus have been thought to be a temporary storage of fertilizing spermatozoa and the release of capacitated spermatozoa a few at a time to ensure that oocytes in the ampulla are not swarmed by excessive spermatozoa [15, 25]. In human females, a specific sperm storage site has not been identified [26].

The isthmus is a major segment of oviduct; it secretes many molecules that affect the physiology of spermatozoa, oocytes, and developing preimplantation embryos. Since the oviduct undergoes very active adovarian peristaltic movements during the periovulatory period (see movies presented by Hino and Yanagimachi [27]), all molecules secreted by the isthmus epithelium must be “pumped up” toward the ampulla before, during, and even after ovulation. Therefore, all of the spermatozoa and oocytes within the oviduct must be “bathed” by molecules secreted by the isthmus epithelium. Isthmus secretions seem not only to render oocytes and spermatozoa more interactive but also to facilitate the development of preimplantation embryos [28–31].

**Figure 2:**
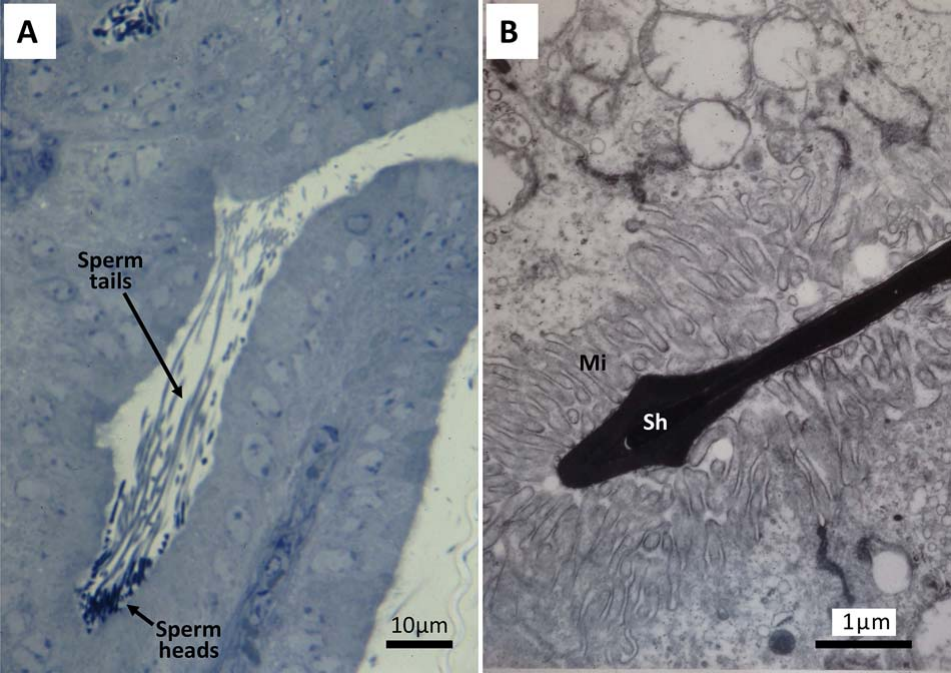
Spermatozoa of golden (Syrian) hamster (*Mesocricetus auratus*) in the oviduct isthmus after natural mating. (A) A group of spermatozoa in a pocket formed by two mucosal folds. (B) Darkly stained sperm head (Sh) appearing to be trapped by microvilli (Mi) of epithelial cells.

It is important to note that the mucosal epithelial cells of the isthmus epithelium are very fragile. They can be readily disrupted by manipulating or flushing the oviduct; therefore, the collection of isthmus secretions must be done very carefully, or collected samples could be contaminated by fragments of the plasma membrane and various intracellular components released from disrupted epithelial cells. Such contaminations could also include “oviductosome-like particles,” which might be formed as artifacts of manipulation, rather than through physiological secretory processes. True oviductosomes are membrane-bound vesicles produced by the process of apocrine secretion [32, 33]. Figure 1 shows an electron micrograph of the isthmic region of mouse oviduct at about the time of ovulation after natural mating. The fluid within the oviduct is by no means crystal clear. It contains many amorphous, granular, and vesicular materials. Much more studies are needed to learn about the origin, nature, and function of materials present in the oviduct before and during fertilization. Combination of biochemical, molecular, and ultrastructural investigations is needed to better understand the micro-environment within the oviduct before and during fertilization in vivo. Figure 2A and B shows light and electron micrographs of golden hamster spermatozoa in the oviduct isthmus after natural mating. Whether sperm contact with microvilli of isthmus epithelial cells is just physical contact between the two cell types or indicates some material exchanges between cells remain to be investigated.

Surplus spermatozoa, if not all, are phagocytized by epithelial cells of the lower segment of the isthmus [34–36], not by cells of other segments of the oviduct. It is puzzling why spermatozoa are phagocytized there instead of draining to the uterus and vagina. What happens to cumulus cells? Also phagocytized?

## 3. Sperm chemotaxis

It is tempting to speculate that eggs or cumulus cells secrete chemoattractants to guide spermatozoa from the lower segment of the oviduct to the upper segment where oocytes await the arrival of spermatozoa. Do oocytes or the oocyte-cumulus complex secrete a sperm attractant that diffuses from the ampulla down to the isthmus where spermatozoa reside before ascending to the ampulla? This is most unlikely, at least in common laboratory animals (e.g., hamster and mice), because oviducts exhibit a very active, adovarian peristalsis which brings the fluid within the oviduct upward (toward the ampulla) rather downward during the periovulatory period [27, 37]. It is important to note that the oviduct’s peristalsis is very sensitive to temperature and dehydration. We have observed that when oviducts are exposed to lower temperature or to the drying effect of air that may occur during surgery or when they have been removed from the female, peristalsis usually slows down and stops.

After spermatozoa have reached the ampulla, will eggs attract spermatozoa? It is known in the mouse and rat that cumulus cells actively secrete progesterone even after ovulation [38–40]. Progesterone can not only induce the acrosome reaction of spermatozoa [41–44], but it is also thought to attract spermatozoa chemotactically [45–47]. It is important to know whether progesterone concentration in the cumulus oophorus (CO) is the highest around the oocyte and the lowest in the periphery of the cumulus. Certainly, cumulus cells near the mouse egg are packed more tightly than those in the periphery of the cumulus (see Figure 2 of [48]), but it has not been demonstrated that there is a progesterone concentration gradient within the cumulus matrix.

It is known that a small fraction of the cortical granules (CGs) in the egg cortex are extruded from the oocyte cortex during the first polar body formation [49, 50]. One wonders whether CG material diffuses outward into the cumulus matrix through the zona pellucida, thus producing a concentration gradient of CS material in the cumulus matrix to direct spermatozoa toward the egg. This is a purely speculative proposal.

It is possible that the cumulus directs spermatozoa to the egg physically rather than chemically. In a fully mature mouse cumulus-oocyte complex, for example, cumulus cells near the egg are arranged radially (see Figure 3 of [28] and Figure 2 of [27, 51, 52]). In other words, there are many radially arranged cell-free, matrix-filled “canals” in the cumulus. Spermatozoa entering the CO, if not all, may pass through these canals to reach the egg. Perhaps, a radial arrangement of cumulus cells directs spermatozoa toward the oocyte physically rather than chemically.

When an egg ages within the oviduct, the CO disintegrates gradually and egg becomes “naked or almost naked''. Some of these eggs, if not all, are still fertilizable. How are these eggs fertilized? It is unlikely that naked eggs secrete progesterone to attract spermatozoa. It must be random collision of spermatozoa and oocytes in the fluid of oviduct, which is constantly moving forward and backward by peristalsis of the oviduct [27]. As long as eggs are able to develop into healthy offspring, fertilization without participation of cumulus cells should be considered normal.

There are numerous papers reporting chemotaxis, rheotaxis, and thermotaxis of mammalian spermatozoa. The authors of these papers maintain that spermatozoa in the lower segment of oviduct move upward being guided by (1) a concentration gradient of substances secreted by the egg, cumulus, or oviduct ampulla—chemotaxis, (2) fluid flow from the ampulla to the isthmus—rheotaxis [52, 53], and (3) a temperature gradient slightly higher in the ampulla than the isthmus—thermotaxis [54, 55]. As already mentioned, oviducts of laboratory rodents display a very active, adovarian peristalsis during the periovulatory period. This oviduct movement brings fluid inside of the oviduct upward (from the isthmus to the ampulla) rather than downward (from the ampulla to the isthmus). At least in rodents, the movement of spermatozoa from the lower to the upper segments of oviducts is very likely neither chemotactic, thermotactic, nor rheotactic. It is unknown whether oviducts of larger mammals, including humans, display an active, adovarian peristalsis during the periovulatory period, but at least in the rabbit an active peristaltic movement of oviducts within the body cavity was observed [56].

## 4. Problems of sperm capacitation

Capacitation is referred to as a process that makes spermatozoa capable of fertilizing zona pellucida-enclosed eggs without any delay. It naturally takes place within the female genital tract, but it can occur under proper in vitro conditions. Since capacitation commonly takes hours, not seconds to complete, it must involve many physical and/or chemical events that occur slowly. The release/removal of decapacitation factors of epididymis and seminal plasma origin [57, 58] as well as the removal of cholesterol [59, 60] and beta-defensin [61, 62] from the sperm plasma membrane are just few examples of many events that are believed to occur during capacitation.

**Figure 3:**
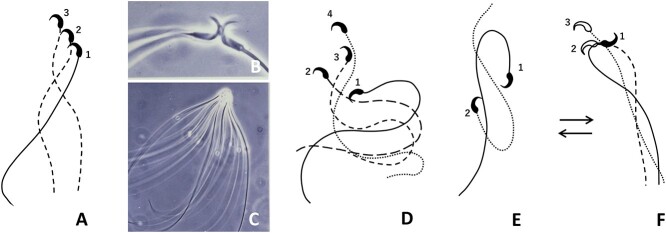
Behavior of golden hamster spermatozoa in fertilization-supporting medium. (A) The flagellar beating pattern of epididymal spermatozoon shortly after release into medium. Note the shallow, symmetrical waves that originate near the head (labeled 1, 2 and 3). Spermatozoa first move freely in the medium, but soon agglutinate head to head, beating their tails stiffly. (B) Two sperm attach to each other by the acrosomal region of the head. (C) A large group of sperm agglutinated head to head. (D, E) After a few hours in capacitation medium, spermatozoa become free from agglutination and swim vigorously, entering the state called “hyperactivation.” Note the deep flagellar bends. (F) When hyperactivated sperm enter a viscous medium or cumulus oophorus, they display a serpentine movement. When they return to nonviscous medium, they resume hyperactivated (“jumping around”) motion (E).

Figure 3 illustrates the behavior of golden hamster spermatozoa in a culture medium and after they become capacitated in vitro. When spermatozoa from the cauda epididymis are suspended in a fertilization-supporting medium (m-TALP), they at first swim individually (Figure 3A) but soon they agglutinate head-to-head (Figure 3B). The number of spermatozoa in each agglutination varies from 2 to almost 100 but is commonly 7–20 (Figure 3C). All spermatozoa beat their tails stiffly without much bending. This state lasts about 2 h. Then, spermatozoa become free from agglutination one by one and swim vigorously by flexing their tails (Figure 3D). They soon change their swimming pattern to display so-called hyperactivated motility (Figure 3E). When a hyperactivated spermatozoon enters a “viscous” medium (or CO matrix), it displays a serpentine movement (Figure 3F). When it returns to a nonviscous medium, it resumes a “jumping around” motion.

Apparently, characteristics of the sperm plasma membrane change dramatically during capacitation. It seems that something covering the plasma membranes of the sperm head and tail has been removed or modified during these 2 h in the case of golden hamster. It is important to emphasize that spermatozoa collected directly from the epididymis or semen are simply unable to enter the egg’s vestments without incubation under capacitating conditions [4, 63].

We should remind ourselves that what happens in vitro may not happen in vivo. For example, mouse spermatozoa in vitro undergo the acrosome reaction on the surface of the oocyte’s zona pellucida [64, 65], but spermatozoa in the oviduct seem to begin the reaction while they are in the isthmus [48, 66]. We must reinvestigate where spermatozoa of many other species begin their acrosome reaction in vivo.

Capacitation is defined as a process through which a sperm gaining the ability to fertilize oocytes immediately. Since mammalian spermatozoa take at least 5–20 min or more to cross the zona pellucida [67–69], only sperm samples capable of fertilizing *all or almost all* of normal eggs within 30 min (or 60 min at most) in vitro or after putting them near the eggs in vivo should be considered “capacitated.”

Capacitation normally takes place within the female genital tract [70–73], but it can take place within various artificial media. We must be aware that no single medium capacitates spermatozoa of all species of mammals. Spermatozoa of some species (e.g., mouse, rat, guinea pig, and human) can be capacitated in ordinary embryo culture media, but those of some other species require the presence of additional specific substances in the capacitation medium: heparin for bovine spermatozoa [74] and neurotransmitter and antioxidant (epinephrine and taurine) for golden hamster spermatozoa [75] for example. This variation is understandable as the internal environment of the female genital tract of a species has evolved independent from that in all other species.

It has been thought that tyrosine phosphorylation of sperm proteins is a key component of capacitation [76, 77]. Proposed sites of tyrosine phosphorylation include the sperm plasma membrane above the acrosomal cap [78], the outer acrosomal membrane [79], fibrous sheath, dense fiber, and axoneme of the sperm tail [80–82]. It was unexpected that the tyrosine kinase inhibitor PF431396 did not prevent spermatozoa from becoming fertilization competent [83], even though it reduces tyrosine phosphorylation. The function of sperm protein phosphorylation may just be to maximize the efficiency of spermatozoa to fertilize. We should be aware that mouse spermatozoa maintained in the medium with H-89 (a potent C-protein kinase A inhibitor) do not undergo protein tyrosine phosphorylation, yet they become capable of undergoing both the acrosome reaction and hyperactivation to fertilize cumulus-enclosed eggs [84]. Thus, at least in the mouse, spermatozoa can become “capacitated” without protein tyrosine phosphorylation. Other types of protein phosphorylation may be involved in sperm capacitation.

Guinea pig spermatozoa with very large acrosomes are interesting in that they are able to undergo both the acrosome reaction and hyperactivation without preincubation in capacitation-supporting media. Those collected from the cauda epididymis and suspended in a fertilization medium containing low concentrations of membrane-active reagents like Hyamine for 10–15 min begin the acrosome reaction and hyperactivation and fertilize zona pellucida-enclosed eggs very efficiently [85]. Barros et al. [86] first published and I confirmed that guinea pig spermatozoa undergo the acrosome reaction when they are compressed under a coverslip. The procedure I used was simple: Spermatozoa from the cauda epididymis are suspended in a simple sperm incubation medium (102.3 mM NaCl, 1.7 mM CaCl_2_, 25.1 mM NaHCO_3_, 0.25 mM Na-pyruvate, 21.5 mM Na-lactate, and 5.5 mM D-glucose). Next, a tiny drop of the sperm suspension is placed on a glass slide and covered with a medium-sized coverslip (on warm microscope stage). This compresses spermatozoa under the coverslip. After 20–30 s, a large drop of culture medium is added to the periphery of coverslip to allow spermatozoa to move freely in the medium. Many spermatozoa swim vigorously (hyperactivated). Many of them are acrosome-reacted. When mixed with zona pellucida-enclosed eggs, the spermatozoa were able to fertilize (Yanagimachi, unpublished data). According to Green [87], 95% of guinea pig epididymal spermatozoa undergo the acrosome reaction within 10 min after Ca^2^^+^ ionophore treatment. Thus, at least in the guinea pig, mature spermatozoa leaving the male’s body are ready to undergo both the acrosome reaction and hyperactivation without any preincubation (capacitation). Something covering the sperm surface seems to be preventing spermatozoa from undergoing the acrosome reaction and hyperactivation. The removal or alteration of this coat seems to be the essence of capacitation.

Tyrosine phosphorylation of sperm proteins believed to be an essential component of capacitation takes place in the plasma membrane covering the acrosome of sperm head as well as the fibrous sheath, dense fibers, and axoneme in the principal piece of sperm tail where the Ca^2+^ channel protein CatSper is localized [80, 88, 89]. The ubiquitin-proteasome system, which plays critical roles in sperm acrosome reactions and sperm-oocyte interactions [90], may not be involved directly in sperm capacitation [91]. It is rather astonishing that sperm capacitation, discovered 70 years ago [2–4], still remains a bit of a mystery today.

Metaphorically, the spermatozoon is like a planetary rocket that makes a one way trip of no return. Its mission is to deliver the nucleus (astronaut) to an egg (planet) covered by very thick clouds (cumulus and zona pellucida). Although a spermatozoon has ample storage of energy (fuel), it absorbs some energy from its environment (the female tract) as a rocket may use both stored energy (fuel) and solar energy. It is most unlikely that the rocket (spermatozoon) carries a factory (machinery) to build new structural components after launching (leaving the male’s body). Switching on and off of a built-in instrument must be the major task that the spermatozoon (rocket) does during its trip. Readers are referred to du Plessis et al. [92] and Xu et al. [93] for the process and mechanisms by which spermatozoa generate energy needed for their survival and functions.

## 5. Sperm hyperactivation

As stated previously, cauda epididymal hamster spermatozoa released into a fertilization-supporting medium first showed a slow tail beating. Several hours later, they were moving very fast and beating their tails vigorously. We saw spermatozoa showing a similar vigorously movement within oviducts of mated female hamsters [94, 95], which I initially called “activation” of spermatozoa. Later, I coined the term “hyperactivation” [96] after consultation with Dr. C. R. Austin [25] because the term “activation” had already been used to refer to the initiation of sperm movement when quiescent spermatozoa in the epididymal and vas deferens begin to move on contact with the seminal plasma or physiological salt solutions.

Since then, the occurrence of sperm hyperactivation was confirmed in various other mammals including the human (for reviews, see [25, 97]). The principal roles of sperm hyperactivation are believed to be: the release of fertilizing spermatozoa from the mucosal folds in the oviduct’s isthmus which serve as a reservoir of fertilizing spermatozoa [98, 99], and enhancement of sperm passage through the viscous CO matrix [100] as well as the semi-solid zona pellucida of the egg [95, 101]. Although the power output of a spermatozoon before and after hyperactivation is about the same, the large tail undulations of a hyperactivated spermatozoon provides a maximal thrust against objects, such as the CO and the zona pellucida [102].

The initiation and maintenance of sperm hyperactivation require Ca^2+^ [103]. When hyperactivated hamster spermatozoa were washed with Ca^2+^-free medium and examined 5 min later, they were still hyperactivated (“jumping around”). Then, 30 min later, they were all still motile, but none were hyperactivated. They were moving rather sluggishly. On the addition of Ca^2+^ (1.8 mM) to the medium, hyperactivated motility of spermatozoa was restored. Ren et al. [104] first reported that sperm hyperactivation is mediated by a specific Ca^2+^ channel protein called CatSper in the plasma membrane of the sperm tail. CatSper-null spermatozoa are motile but are unable to exhibit hyperactivated motility and are unable to pass through the egg’s zona pellucida to fertilize [89, 99, 104, 105].

One thing that we should be aware of is that, at least in the mouse and guinea pig, mature spermatozoa collected from the cauda epididymis and vas deferens are able to exhibit both the acrosome reaction and hyperactivated motility without “capacitation.” For example, guinea pig spermatozoa collected from the cauda epididymis and suspended in an ordinary bicarbonate-buffered balanced salt solution with 0.003% detergent Hyamine 2389 underwent both hyperactivation and the acrosome reaction within 15 min. They were able to fertilize zona pellucida-enclosed eggs [85]. According to Barros et al. [86], guinea pig spermatozoa compressed between a slide and coverslip for a few minutes underwent the “acrosome reaction.” Those recovered by running medium under the coverslip were able to fertilize zona-free hamster eggs. I repeated this experiment and found that acrosome-reacted guinea pig spermatozoa thus produced were able to fertilize zona-intact guinea pig eggs (Yanagimachi, unpublished data).

Hyperactivation and the acrosome reaction of spermatozoa are needed for fertilization of normal, zona pellucida-enclosed eggs. Dysfunction of either one of them results in fertilization failure. Normally, sperm hyperactivation begins before the acrosome reaction. After the acrosome reaction, hyperactivated spermatozoa become even more vigorously motile [106]. This makes sense because it is the acrosome-reacted spermatozoa that are able to pass through the “solid” zona pellucida.

Whether sperm head and tail compartments are separated or interconnected in terms of ions is important to know. Can the acrosome reaction occur without sperm tail? Can hyperactivation of the tail occur without a sperm head? These questions may be answered by experiments using mutant mice producing spermatozoa with heads and tails separated, for example, by knocking out gene Spata6 [107]. In normal spermatozoa, the sperm plasma membrane is fixed (fused with?) with the nuclear envelope, at the “posterior ring” (see Figure 3-3 of [26]). Whether the posterior ring plays an important role in ionic communication or separation between sperm head and tail must be investigated. No doubt that both extracellular and intracellular Ca^2+^ play crucial roles in sperm dynamics. Readers are referred to Costello et al. [108] for the dynamics and functions of the sperm’s internal Ca^2+^ store.

## 6. Time, site, and cause of sperm acrosome reaction in vivo and in vitro

Although the acrosome reaction of mammalian spermatozoa has been studied extensively, we still do not have a general consent of the time, site, and cause (inducer) of the reaction. The status of the sperm acrosome seems to be affected by the physiological state of the female in vivo as well as biochemical components in media we use for experiments in vitro. Two things that are certain are (1) uncapacitated spermatozoa with intact acrosomes are unable to enter the CO surrounding the egg and (2) spermatozoa pass through the zona pellucida only after completing the acrosome reaction. In mice, it was thought for a long time that the egg’s zona pellucida induces the acrosome reaction in spermatozoa. In fact, zonae pellucidae of the mouse and many other mammals including humans can induce the acrosome reaction efficiently [64, 109–112]. However, mouse spermatozoa in vivo seem to begin their acrosome reaction while ascending the oviduct from the isthmus to the ampulla where fertilization takes place [48, 66, 113]. Austin and Bishop [114] first reported the presence of acrosome-reacting mammalian (hamster) spermatozoa in the CO. Cummins and Yanagimachi [63] found that hamster spermatozoa with swollen acrosomes (most likely fully capacitated) entered the cumulus and completed their acrosome reaction while passing through the cumulus or soon after reaching the zona pellucida. Acrosome-reacted hamster spermatozoa were unable to enter the cumulus. According to Corselli and Talbot [115] however, acrosome-reacted hamster spermatozoa are able to enter the cumulus, but they are unable to reach the zona. This is in contrast to the report by Inoue et al. [116] that acrosome-reacted mouse spermatozoa are able to pass through the cumulus to reach the zona. In humans, fertilizing spermatozoa in vitro seem to begin their acrosome reaction within the cumulus and complete the reaction on the zona pellucida [117, 118]. Thus, the readers see examples of inconsistent and confusing reports from investigators who used different species and different conditions for their studies.

We investigators are all looking for specific physiological factor(s) that trigger or promote the acrosome reaction of fertilizing spermatozoa. We should remind ourselves that spermatozoa of many species, including humans, can undergo the acrosome reaction spontaneously and fertilize eggs in defined media without the presence of any specific reagents, compounds, eggs, and their coating materials. This does not mean that specific substances are not involved in sperm acrosome reactions in vivo.

There are many reports that oviductosomes secreted from the oviduct contribute to capacitation and the acrosome reaction of spermatozoa in vivo [33, 119, 120]. This will be discussed in the next section.

## 7. Oviductin and oviductosomes: the oviduct’s secretory products

Although in vitro fertilization and subsequent development of preimplantation embryos are now possible in many different species of mammals, we are aware that most of us started our lives within our mothers’ oviducts. Ronald Hunter has been a consistent advocate who has been urging us to appreciate the importance of the study of the mammalian oviduct to better our understanding of what happens there during the beginning of the lives of all mammals including our own species [18, 121, 122].

While one of my former associates and I were working on hamster oocytes before and after ovulation, we noted a distinct difference in the optical property of the zonae pellucidae of oocytes before and after entry into the oviduct. We also noted that the zonae pellucidae of oviductal oocytes have a greater ability to induce acrosome reactions in spermatozoa than those of ovarian oocytes [123]. Oikawa et al. [124] reported the presence of a 200–240 kDa glycoprotein in the hamster oviduct that alters characteristics of the zona pellucida. This molecule, later called “oviductin” [125], binds to the zona pellucida and enhances sperm penetration through the zona [126]. Zhao et al. [127] maintain that human oviductin, which binds to human spermatozoa, potentiates the acrosome reaction. Unexpectedly, oviductin knockout female mice were as fertile as wild-type (control) females [128]. However, it is hasty to conclude that oviductin is not essential for fertilization in all animals and humans, because acrosin-null male mice, for example, are fertile (strictly speaking, less fertile than wild-type males) [129], whereas acrosin-null male hamsters are totally infertile [130]. Whether oviductin-null males of other mammals are fertile, subfertile, or infertile must be investigated. There are many excellent reviews on the roles of oviductal secretions in gamete physiology, gamete interactions, and preimplantation embryo development [28, 29, 131, 132].

Recently, much attention has been directed to “oviductosomes” secreted by epithelial cells that line the oviduct lumen. They are nano-size protein and mRNA-containing vesicles that bind to (and fuse with) spermatozoa to facilitate capacitation and fertilization [120, 133–135]. Although some of the vesicles reported by these authors seem to be secreted by the oviductal epithelium, some proteins like Ca^2+^-ATPase [136] are very likely released from epithelial cells disrupted during the flushing of oviducts with medium. The oviduct’s mucosal epithelium, in particular that of the isthmus, is delicate and can be readily disrupted by harsh handling of the oviduct.

## 8. Size and shape of spermatozoa

**Figure 4:**
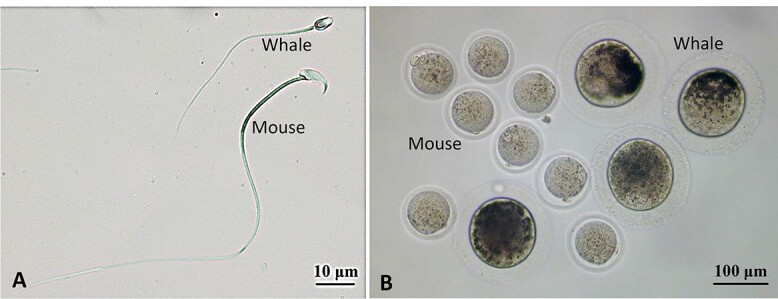
Spermatozoa (A) and mature oocytes (B) of the mouse and the Bryde’s whale (*Balaenoptera brydei*). This whale is 12–15 m in body length. These photos were provided by Drs. Yutaka Fukui and Hiroyuki Watanabe.

Whales and cattle have much larger oviducts and uteri than humans and mice (Table 1). While spermatids of mice, humans, and whales are similar in size, mouse spermatozoa are much larger (longer) than human and whale spermatozoa (Figure 4). It is the length of the tail (flagellum), not the head (nucleus), that makes the difference [137].

**Table 1:** Comparison of approximate sizes of female genital tract, fully mature oocytes, and spermatozoa of the mouse, human, and whale.

	Mouse	Human	Whale^*^
Oviduct: length, stretched	1.5 cm	12 cm	40 cm
Uterus: length	1.5 cm	10 cm	70 cm
Oocyte proper: vitellus diameter	75 μm	120 μm	120 μm
Zona pellucida: thickness	7.5 μm	20 μm	30 μm
Round spermatid: diameter	10 μm	10 μm	10 μm
Sperm: entire length (head length) ^**^	120 μm (8 μm)	50 μm (4 μm)	60 μm (5 μm)
Erythrocyte:	7.3 μm	7.8 μm	7.7 μm

^*^Bryde’s whale (*Balaenoptera brydei*), 12–15 m in adult body length. Length of oviduct and uterus was estimated by Dr. Hiroyuki Watanabe.

^**^Note that mouse sperm head is “flat,” whereas the heads of human and whale sperm have a more rounded shape.

It is generally assumed that sperm swimming velocity is positively associated with the length of spermatozoa and that males with longer spermatozoa have advantages in fertilization over those with shorter spermatozoa when males compete for mating and the female accepts more than one male [138, 139]. This does make sense if the female genital tract is motionless, and its inner wall is uniformly smooth. However, the oviduct is not a simple straight, stationary tube. In the mouse, for example, the inner wall of the oviduct has numerous folds and pockets and the oviduct itself manifests very active, peristaltic movements during the periovulatory period (see movies of the mouse oviduct with very active peristalsis (Hino and Yanagimachi [27], see Supplemental movies of oviduct’s movement). Spermatozoa stick to and detach from the epithelium that lines the oviduct lumen before reaching the ampulla where fertilization takes place. It must be both the adovarian peristaltic movement of the female tract and the sperm’s own movement that brings spermatozoa to the ampulla of the oviduct. As seen in Table 1, round spermatids of the mouse, human, and whale are all about the same size; therefore, there must be some genetic control of the length of the sperm tail. If this gene(s) is identified, then we should be able to produce mutant animals with spermatozoa larger or smaller than normal (wild-type) spermatozoa. It would be of great interest to see which has advantage over the other in fertilizing oocytes in the oviduct.

The spermatozoon is composed of a head and a tail. What determines the shape, length, and volume of these two structures is still totally unknown ever since Fawcett et al. [140] speculated that the form of the sperm head is probably determined not by external force (e.g., from the Sertoli cell) but by DNA and nuclear protein during chromatin condensation. Today we have many molecular and genetic tools to study sperm morphogenesis. It is hoped that this age-old problem of the sperm’s dimensional variation is solved before long. If we can control the size and shape of spermatozoa, we will learn much more about the physiology and competition of spermatozoa.

## 9. Roles of protease/proteasomes in sperm capacitation and sperm-oocyte interaction

The acrosome contains various hydrolyzing enzymes [25, 141]. Hyaluronidase and acrosin are the ones that have been studied most extensively. They are believed to be important for the sperm acrosome reaction as well as the passage of spermatozoa through the CO and zona pellucida surrounding the oocyte. It is Sutovsky and his associates who played a leading part in disclosing the important role of sperm proteasomes in various steps of mammalian fertilization [90, 142]. It is most likely that proteases including acrosin and proteasomes work synergistically in various steps of fertilization, including sperm capacitation, the acrosome reaction, sperm passage through the zona pellucida, and even the egg’s block to polyspermy. This will be discussed separately.

## 10. Roles of acrosomal enzymes in fertilization

Spermatozoa of all mammals have acrosomes. Although the shape and size of the acrosome varies greatly from species to species, its fundamental structure is the same. It is composed of the anterior thick acrosomal cap and the posterior, thin equatorial segment. While the acrosomal cap contains a variety of hydrolyzing enzymes, the equatorial segment is believed to be devoid of enzymes [25].

Hyaluronidase is the acrosomal enzyme first discovered and well characterized. Although it can depolymerize the gelatinous matrix of the CO, its role in fertilization has been the subject of controversy. Mouse spermatozoa have two kinds of hyaluronidases, cell surface hyaluronidase (e.g., SPAM_1_/PH20) and intra-acrosomal hyaluronidases (e.g., HYAL5). Surprisingly, mouse spermatozoa without these two hyaluronidases are fertile, but their spermatozoa are less fertile than normal (wild-type) ones due to their inferior ability to enter or pass through the cumulus [143, 144]. Perhaps, there are many other fertility-related acrosomal enzymes that are not essential, but their presence enables the proceeding of important steps when the primary enzyme does not work well.

Acrosin, another well-characterized acrosomal enzyme, is important for the swelling and dispersion of the acrosome inner matrix [145–147] as well as sperm penetration through the oocyte’s zona pellucida, at least in the hamster [130]. In the mouse, we can prepare nearly 100% acrosome-reacted, live spermatozoa by preincubating epididymal spermatozoa in capacitation medium for 2 h then treating them with Ca^2+^ ionophore [148]. It would be interesting to know whether such spermatozoa are able to attach to and penetrate the zona pellucida in the presence of proteinase inhibitors such TLCK, benzamidine, and soybean trypsin inhibitor.

**Figure 5:**
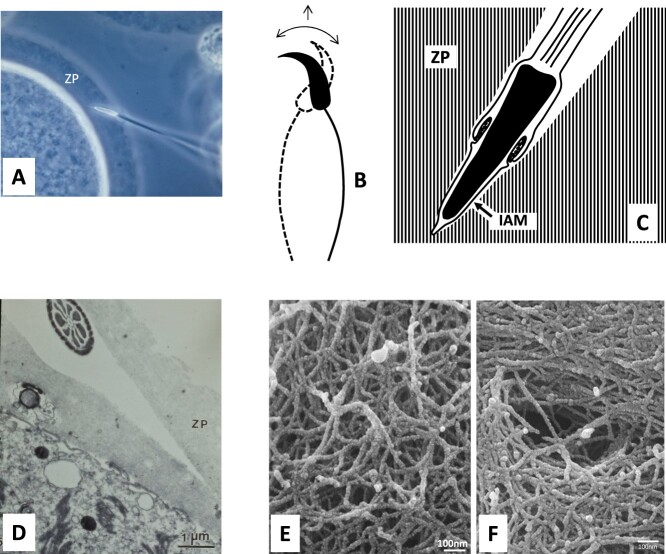
Sperm passage through the zona pellucida (hamster). (A) Light microscope image of a live spermatozoon penetrating through the zona pellucida (zp). This egg was lightly compressed between slide and a coverslip before photographed and therefore the PVS between the zona and egg proper had disappeared. (B) Spermatozoon advances through the zona by a scything motion of its head. (C) Each spermatozoon leaves a sharply defined canal (“ penetration slit”) in the zona. Zona pellucida (ZP) and inner acrosomal membrane (IAM). (D) Electron micrograph of a penetration slit. A cross section of sperm flagellar midpiece of sperm is seen in the slit. (E, F) Scanning electron micrographs of human zona pellucida, showing its fibrous network. A “hole” in the fibers of zona (F) representing a canal through which a follicle cell near the oocyte inserted its process to give nourishment to the growing oocyte. Scanning electron micrographs of human zona pellucida (E and F) are from Familiari et al. [383].

When we watch a spermatozoon passing through the zona pellucida (Figure 5A), we see the sperm head advancing forward a little at a time by a scything motion of the head (Figure 5B). The sperm head leaves a “canal” with sharp contour (Figure 5C and D). This gives the impression that the sperm head cuts open the zona matrix mechanically. However, the surface of the sperm’s inner acrosome membrane may be “covered” by membrane-anchored acrosin, which serves as a “lubricant” of sperm head passage through the zona. Under the light microscope, the zona pellucida appears as a homogeneous gelatinous material, but it is actually made of a mucopolysaccharide network (Figure 5E and F).

It is important that the acrosome reaction makes spermatozoa capable of fusing with oocyte’s plasma membrane [25]. The acrosomal enzyme acrosin, which is exocytotically released during the acrosome reaction, seems to make the sperm plasma membrane fusion competent [149]. Activation and migration of IZUMO1 (sperm’s gamete fusion-mediating protein) from the outer acrosomal membrane to the plasma membrane of the equatorial segment of the acrosome (see Section 15 on sperm-oocyte fusion) may also require proteolytic activity of acrosomal proteases.

Although there are many papers reporting the presence of acrosin on the inner acrosomal membrane during and after the acrosome reaction [145, 150–152], there are other papers reporting its absence [153, 154]. Further studies are needed to determine which is correct. For localization of acrosin, it is very important to wash live acrosome-reacted spermatozoa thoroughly prior to application of anti-acrosin antibody, or acrosomal matrix with acrosin activity might precipitate on the inner acrosomal membrane during sample preparation.

Yudin et al. [155] maintained that the inner acrosomal membrane of macaque spermatozoa has PH-20 with hyaluronidase activity and this, not acrosin, plays the essential role in zona penetration by spermatozoa. One should be aware that hyaluronic acid is present in the outer half of the zona pellucida as well as in the cumulus matrix [156].

Sutovsky has been the leading advocate of the importance of sperm proteasomes in various steps of mammalian fertilization such as sperm capacitation, the acrosome reaction, and zona penetration by spermatozoa. Proteasomes are on the outer and inner acrosomal membranes as well as within the acrosome. Those on the outer acrosomal membrane and acrosomal matrix are likely involved in the acrosome reaction; those on the inner acrosomal membrane may play important roles in sperm head attachment to and penetration though the zona [90, 142, 157–160]. Sutovsky [90] maintained that proteasomes are on the inner acrosomal membrane, which makes direct contact with the zona pellucida. However, electron micrographs presented as evidence (Figure 7 of Sutovsky et al. [157]) are not convincing to verify the presence of proteasomes on the inner acrosomal membrane. To locate the site of “zona lysin” candidate(s) within spermatozoon, light microscopy is not appropriate because the sperm nucleus (head) is covered by various membranes: the plasma membrane, the outer and inner acrosomal membranes, and the nuclear envelope. Light microscopy cannot differentiate among these membranes. To demonstrate zona lysin on the inner acrosomal membrane, live acrosome-reacted spermatozoa must be washed thoroughly before fixation. Fixation of spermatozoa during the acrosome reaction is not recommended because the contents (matrix) of the acrosome may precipitate on the inner acrosomal membrane as well as the plasma membrane covering the rest of sperm head.

## 11. Why is the presence of cumulus oophorus (CO) around the egg beneficial for fertilization?

Mammals are unique in that ovulated eggs are each surrounded by nursing cells which provided nutrients to the growing oocytes. These cells and their matrix are collectively called the CO. Although fertilization is possible without CO, the presence of CO around each oocyte seems to increase the chance of successful fertilization at least in vitro [161–163]. Hyaluronic acid in CO may enhance the zona pellucida’s ability to induce the acrosome reaction of spermatozoa [164]. Induction of human sperm acrosome reaction by CO matrix has been reported repeatedly [165, 166]. Bedford and Kim [167] postulated that expanded CO fills the lumen of the oviduct ampulla and “traps” spermatozoa ascending from the lower segment of the oviduct. In some animals, progesterone secreted by CO cells may trigger the acrosome reaction of spermatozoa. Why cumulus-enclosed oocytes are fertilized better than less cumulus-free ones could be explained in physical terms. The presence of the viscous CO matrix around the oocyte prevents or reduces rotation of the egg while a spermatozoon, with its head inserted into the zona pellucida, beats it tail vigorously. The tip of the acrosome-reacted spermatozoon is sharply pointed and its scything motion cuts open the zona matrix [67, 168]. The presence of a “viscous” cumulus matrix around the sperm tail would provide more thrusting power to the sperm head than when spermatozoa are in a low viscous medium [169]. If this assumption is correct, cumulus-free eggs held by a capillary pipette would be fertilized more readily than those free in the medium. Such an experiment is yet to be done.

## 12. How do spermatozoa pass through the zona pellucida of eggs?

Field vole spermatozoa are very interesting. As expected, acrosome-reacted spermatozoa pass through the zona pellucida of the egg of its own species, but vole spermatozoa are able to go through the zona of a mouse egg without the acrosome reaction [170]. Since field vole spermatozoa swim very fast (author’s unpublished observation), acrosome-intact vole spermatozoa must cut open the mouse zona mechanically. Perhaps, the zona pellucida of the field vole is much more solid than that of the mouse, but this must be confirmed by actual measurement.

A golden hamster spermatozoon passing through the zona pellucida (Figure 5A) displays a scything motion of its head (Figure 5B) [67, 169]. Human sperm heads also show a similar, scything motion during passage through the zona [168]. Since the “sperm penetration slit (s)” left in the zona pellucida has a sharply defined tunnel (see Figure 5C and D), it is possible that the head of an acrosome-reacted spermatozoon with a sharp-pointed anterior edge cuts through the zona’s glycoprotein network (Figure 5E and F) mechanically. However, it is more likely that spermatozoa use both mechanical and enzymatic means in passing through the zona (cf. see Section “Role of acrosomal enzymes in fertilization”).

It has been thought for many years that the acrosomal protease, acrosin, plays a leading role in sperm passage though the zona. Most of the acrosin is within the matrix of the acrosome, but some is believed to remain on the inner acrosomal membrane after the acrosome reaction. It is this acrosin that is thought to “digest” zona glycoproteins. The finding that transgenic mouse spermatozoa without acrosin are still able to pass through the zona pellucida [129] cast doubt that acrosin is necessary for zona penetration. However, it should be noted that acrosin-null spermatozoa are less efficient at fertilizing oocytes than those of wild-type mice [171]. More recently, it was found that acrosin is indeed needed for hamster spermatozoa to pass though the zona. Acrosin-null hamster spermatozoa are able to undergo the acrosome reaction and attach to the zona pellucida, but they are unable to pass through it [130]. Perhaps, proteasomes [157, 172–174] and acrosin work synergistically during the acrosome reaction and zona penetration.

Intracellular localization of acrosin and proteasomes must be done at the electron microscopic level because the sperm head has several membranes: the plasma membrane, the outer and inner acrosomal membranes, and the nuclear envelope. To see if the inner acrosomal membrane carries acrosin and/or proteasomes, live acrosome-reacted spermatozoa must be washed thoroughly before fixation, or acrosin/proteasome in the acrosomal matrix may precipitate on the inner acrosomal membrane during preparation of sperm samples for electron microscopy. None of the studies published thus far demonstrated convincingly that acrosin or proteasomes are on the inner acrosomal membrane.

Spermatozoa of many species (e.g., hamster, rabbit, guinea pig, pig, sheep) pass through the zona pellucida obliquely (e.g., see Dickmann and Dziuk [175]). Bedford [176] tried to explain why it must be that way. However, rat and human spermatozoa can penetrate the zona perpendicularly [69, 177]. Dickmann and Dziuk [175] saw a thin “process” in front of the pig sperm head within the zona and thought that it might be “homologous” to the acrosomal filament, an extension of the inner acrosomal membrane of invertebrate spermatozoa that develops immediately before fertilization. I witnessed that the head of hamster spermatozoa in the zona pellucida advance forward by a scything motion as already mentioned, but sometimes the head moves backward before moving forward again. The fine process Dickmann and Dziuk [175] saw in front of pig spermatozoon in the zona (see Figure 1 of their paper) must be the slit made by the spermatozoon that had moved backward temporarily.

## 13. Is the zona pellucida essential for fertilization and embryo development?

The answer is no. At least in the mouse, oocytes freed from zonae pellucidae can be fertilized monospermically in vitro and develop into blastocysts that are able to develop into normal offspring after transfer to their own or surrogate mothers [178]. It is important to note that zona-less eggs are prone to become fertilized by more than one spermatozoa (polyspermy) and that zona-free cleaving stage embryos (1–4 cell stages) lost quickly from oviducts after transfer [179]. Modlinski [180] observed “naked” mouse blastomeres adhering to isthmus epithelium before being lost. No one knows whether blastomeres are phagocytized by the oviduct’s epithelial cells or drain into lymphatic lacunae of the isthmus. Unlike human, mouse and most animals do not or seldom have ectopic pregnancy [181].

Apparently, the zona pellucida is important for the protection of the early embryo inside from danger of adhesion to epithelium or other cleaving embryos. In the mouse and perhaps in other mammals and humans, zona-less embryos in the early cleavage stages within the oviduct are trapped by the oviduct’s epithelium and seem to perish. No one has followed the fate of these zona-less embryos carefully. The zona pellucida is essential in vivo, but not in vitro. For in vitro, this is a good example of “far better to have than not have.”

## 14. The presence of a perivitelline space in unfertilized eggs: a unique feature in mammals

In most animals, fully mature eggs are each tightly surrounded by an acellular coat called the vitelline envelope (=zona pellucida). There is no space between the two. They are separated after fertilization or egg activation to create the perivitelline space (PVS). The PVS is formed by colloidal pressure of CG materials released from the egg under the overlying vitelline envelope. Mammals are exceptional in that a PVS exists before fertilization. If there were no PVS in mammals and the egg’s plasma membrane was in close contact with the zona pellucida, fertilization would never occur, because the anterior half of the head of an acrosome-reacted spermatozoon passing through the zona pellucida is covered by a nonfusogenic inner acrosomal membrane. Consequently, the sperm head would likely be prevented from turning to expose its fusogenic plasma membrane to the egg plasma membrane. The PVS would provide the spermatozoa with the chance to reorient the head and fuse with the egg.

How is the PVS formed before fertilization? A PVS does not exist in the fully grown oocyte at the germinal vesicle stage. CGs are evenly distributed in the oocyte’s cortex. During the first meiosis, some CGs are released from the egg cortex above the meiotic spindle; more CGs are apparently released before the egg reaches the metaphase of the second meiosis [49, 50]. This local “precocious” CG exocytosis is likely responsible for the formation of a small PVS before ovulation. The PVS becomes larger after eggs enter the oviduct. It is well known that global exocytosis of CGs during fertilization is triggered by the release of Ca^2+^ from internal stores [182]. How a local CG exocytosis occurs during egg maturation is not known.

## 15. Membrane fusion of sperm and oocyte

In the golden hamster, the oocyte becomes capable of fusing with spermatozoa during its growth when it is about 20 μm in diameter and microvilli first appear. The capacity for fusion increases as the oocyte grows. It reaches the maximum for fusion at metaphase of the second meiosis when the oocyte’s vitellus is about 70 μm in diameter. The fusion capacity of the oocyte is reduced drastically upon fertilization and lost completely by the eight-cell stage of embryonic development [183]. In the mouse, too, the oocyte becomes fusion competent when it is ~20 μm in diameter. The capacity is lost by the four-cell stage [184].

**Figure 6:**
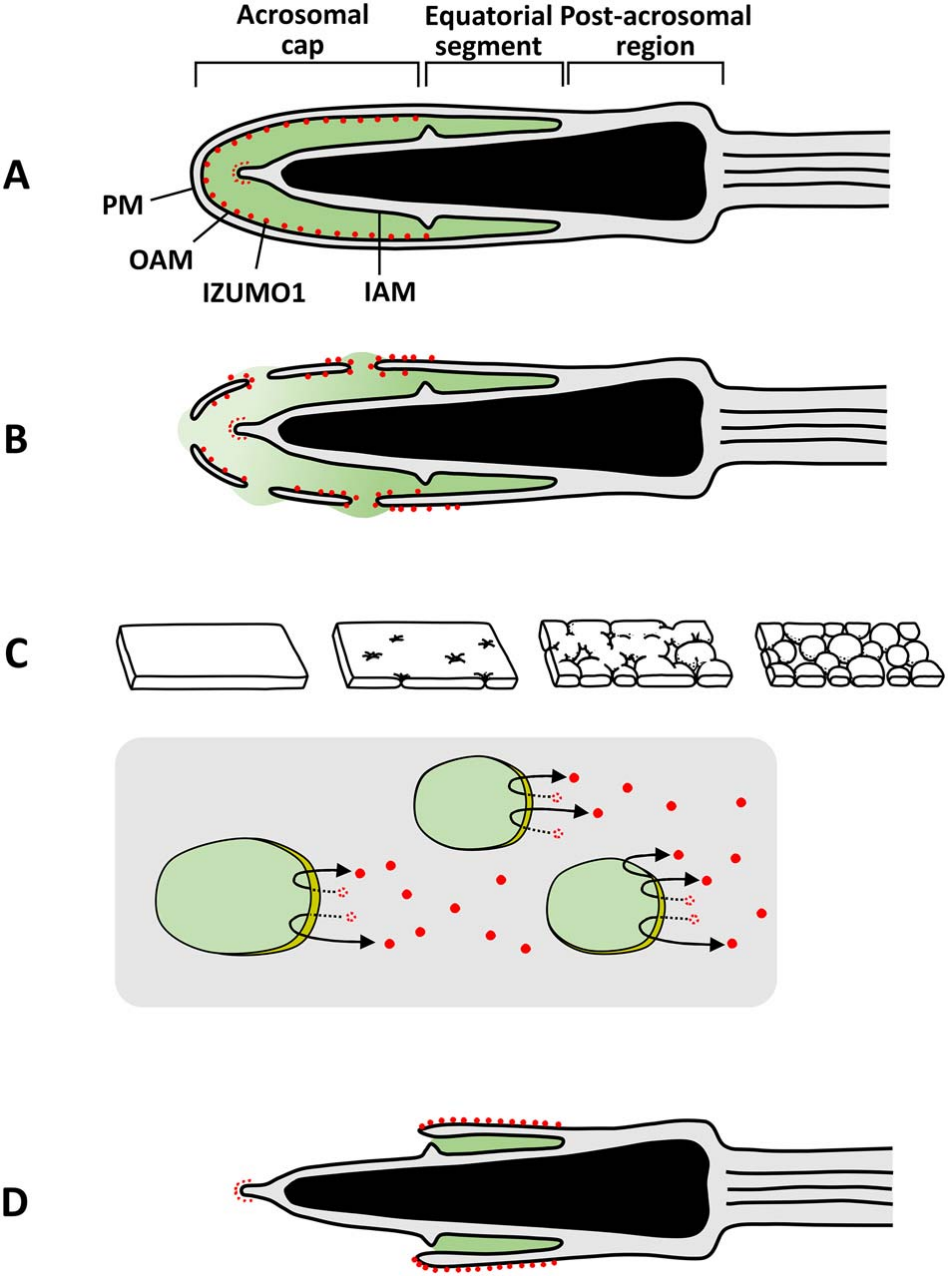
Hypothetical view of IZMO1’s migration during the acrosome reaction. IZUMO1 is shown as a red spot. (A) Before the acrosome reaction, IZUMO1 is on the inner surface of the outer acrosomal membrane. (B, C) During fusion and vesiculation of the plasma membrane with the outer acrosomal membrane, IZUMO1 migrates (diffuses?) via the fusion sites to the plasma membrane of the equatorial segment. Surface views of the plasma membrane of the acrosomal region (C) show how the plasma and outer acrosomal membranes vesiculate and how IZUMO1 may migrate out of the acrosome (acrosome contents shown in green) onto the plasma membrane and toward the equatorial segment. (D) IZUMO1 reaches the plasma membrane of the equatorial segment at the completion of the acrosome reaction. Some IZMO1 are on the inner acrosomal membrane in the frontal edge of the inner acrosomal membrane. They do not change their position during sperm’s acrosome reaction. Four diagrams of membrane vesiculation in the upper row of (C) are from Barros et al. [384].

Unlike oocytes, spermatocytes and even fully mature spermatozoa are unable to fuse with oocytes. Spermatozoa become fusion competent only after completing the acrosome reaction [25, 185]. It is now clear in the mouse that during the acrosome reaction, the membrane protein IZUMO1 quickly relocates from the outer acrosomal membrane to the plasma membrane of the equatorial segment of the sperm head; this relocation makes the spermatozoon fusion-competent [186, 187]. How does IZUMO1 migrate from the outer acrosomal membrane to the plasma membrane above the equatorial segment of acrosome during the acrosome reaction? Figure 6, in my assumption, shows IZUMO1’s migration from the outer acrosomal membrane of the acrosomal cap region to the plasma membrane in the equatorial segment of the acrosome. No one knows whether migration of IZUMO1 is accomplished by a simple lateral dispersion of IZUMO1 molecules in the membrane lipid bilayer or that IZUMO1 migration is aided by actin-based molecular motors such as those involved in the acrosome reaction. Involvement of actin dynamics in the acrosome reaction has been reported [188–192]. The second question is concerned with a temporal migration of IZUMO1 to the plasma membrane of the post-acrosomal region during the acrosome reaction. According to Sebkova et al. [193], IZUMO1 covers the entire surface of sperm head (including the post-acrosomal region) after the acrosome reaction. A supplemental movie prepared by Satouh et al. [194] shows that IZUMO1 quickly spreads over the entire surface of sperm head, then retreats to the plasma membrane of the equatorial segment. Whether or not IZUMO1 migrates to the post-acrosomal region during the acrosome reaction is an important issue. If IZUMO1 does migrate to the post-acrosomal region even temporarily, it may explain why Yanagimachi and Noda [195, 196] and Toshimori [197] saw membrane fusion between the sperm’s plasma membrane of the post-acrosomal region and oocyte’s plasma membrane (microvilli). If IZMO1 does not migrate to the post-acrosomal region, what prevents it from doing so? The third question is whether acrosomal proteinases (e.g., acrosin) are involved in activation and/or migration of IZUMO1. In the hamster, the presence of proteinase inhibitor in the medium during the acrosome reaction and sperm-oocyte interaction markedly reduces sperm’s ability to fuse with oocytes [149]. Acrosomal proteinase may not only induce the swelling of acrosomal matrix (see Section10: Role of Acrosomal Enzymes in Fertilization) but also may contribute to activation and migration of IZUMO1 from the outer acrosomal membrane to the plasma membrane of the equatorial segment region of sperm head. It is highly possible that acrosomal proteasomes work synergistically with acrosomal proteases in this process. As of today, all studies of IZUMO1’s migration were performed at the light microscopic level. It should be done at the electron microscopic level, too, to learn the detail of its migration. The sperm head has many different membranes. Light microscopy does not allow us to follow how IZOMO1 migrates from the outer acrosomal membrane to the plasma membrane of the equatorial segment of sperm head. According to Fusi et al. [198], the adhesion molecule P-selectin appears on the plasma membrane of acrosome-reacted (human) spermatozoa. It is not there before the acrosome reaction. Its origin and functional relationship to IZUMO1 are unknown.

IZUMO1’s counterpart is JUNO, a GPI-anchored membrane protein, on the oocyte’s plasma membrane [199]. It largely disappears from the egg surface after fertilization. Although immune cytochemical micrographs of JUNO presented by Bianchi et al. [199] and Suzuki et al. [200] show that JUNO is on the entire surface of oocyte, those by Mori et al. [201] show no JUNO in the microvilli-free area. It is known that microvilli-free area of the oocyte plasma membrane and the polar body are not capable of fusing with spermatozoa [25]. It should be noted that neither IZUMO1 nor JUNO has fusogenic peptides [202]. They are cell adhesion molecules.

Other presumptive fusion-mediating molecules of oocytes include ITGA9 [203] and CD9. The absence of the former largely reduces the incidence of sperm-oocyte fusion. The latter is required for normal structure of microvilli [204–208]. As of today, sperm molecules other than IZUMO1 that are considered contributing to sperm-egg fusion include equatorin [209], FIMP [210], SOF1, TMEM95 and SPACA6 [211], CRISP2 and DCST1 and 2 [212]. Whether sperm-oocyte fusion is accomplished by collaboration of many different pairs of fusion molecules must be determined. It is possible that the fusion is mediated by a single pair of molecules and many others regulate sperm-oocyte adhesion prior to fusion. Remember that sperm-oocyte fusion is Ca^2+^ and pH-dependent [213, 214]. Why it is Ca^2+^ and pH-dependent must also be investigated. In the mouse and guinea pig, K^+^ must be in media during and after the acrosome reaction to render spermatozoa competent to fuse with oocyte [215, 216]. Its reason also remains unknown.

According to Barros et al. [217], hamster spermatozoa incubated in a medium containing human serum for 4–5 h completely lose the ability to cross the zona pellucida. Equatorial segments of these spermatozoa were extensively vesiculated or lost completely, yet they were able to fuse with zona-free oocytes. IZUMO1 or some unknown fusion-mediating molecules must be in the post-acrosomal region of such spermatozoa.

Another puzzle is the presence of IZUMO1 at the tip of the inner acrosomal membrane (Figure 6A and D) [194]. It is the frontal edge of the sperm head that is “pushed” against zona pellucida as the acrosome-reacted spermatozoon passes through the zona (see Figure 5C). The inner acrosomal membrane never fuses with oocyte’s plasma membrane. Does IZUMO1 at this position act as a zona lysin? After the sperm head passes through the zona pellucida, the tip of the sperm head may (will) touch oocyte’s plasma membrane (microvilli). Does this activate the oocyte? At any rate, the presence and the role of IZUMO1 on the inner acrosomal membrane at the frontal edge of acrosome-reacted mouse spermatozoa are mysteries.

## 16. Sperm-borne oocyte activating factor: it could be spermatid histone

Mammalian oocytes may activate spontaneously during post-ovulatory aging in the oviduct or during in vitro culture. They may also be activated by chemical (e.g., Ca^2+^ ionophore) or physical agents (e.g., electric current). However, it is the spermatozoon that activates an egg under ordinary in vivo and in vitro conditions. Two strong candidates have been proposed as the sperm-borne oocyte activating factors (SOAFs): phospholipase C zeta [218, 219] and post-acrosomal sheath WW-binding protein [220, 221].

**Figure 7:**
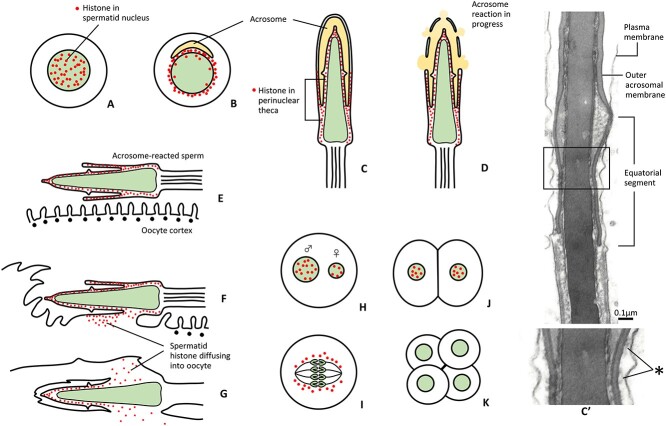
Proposed behavior of sperm histones during spermiogenesis, fertilization, and early embryo development. Spermatid histones are shown as red spots. (A) First, histones are in spermatid’s nucleus (green). (B, C) Histones become part of the sperm’s PNT. (D–G) Spermatozoa during the acrosome reaction and fusion with oocytes. During sperm-oocyte fusion, the histones disperse into the oocyte cytoplasm and activate the oocyte. (H–J) The spermatid histones become incorporated into the male and female pronuclei and are still in the nuclei of 1–2 cell embryos. (K) Spermatid histone no longer exists in the nuclei of four-cell embryo. C′ is an electron micrograph of the sagittal section of the mid-region of rabbit sperm head. Note the presence of amorphous material (*) between the plasma and outer acrosomal membranes of the equatorial segment. This material could be spermatid histone more soluble than those in the post-acrosomal region of sperm head.

Although results of many studies [222–227] seem to support the hypothesis that phospholipase C zeta is the SOAF, I propose that histones in the sperm perinuclear theca (PNT) could be the SOAF. It is purely speculative at this moment but should be taken into consideration. In round spermatids, histone is within the nucleus (Figure 7A). During compaction of the spermatid nucleus, histone is replaced by protamine and released histone becomes incorporated into the PNT (Figure 7B) [228]. In fully developed spermatozoa, sperm histone is a major component of sperm’s PNT (Figure 7C). Part of histone in soluble form could be located between the outer acrosomal membrane of the equatorial segment and the overlying plasma membrane. While Tovich and Oko [228] thought that sperm histone would stabilize the pronuclear development of the sperm nucleus, I propose that PNT histone remains around the sperm nucleus during the acrosome reaction (Figure 7D) and enters the oocyte to activate the oocyte (Figure 7E–G). Kimura et al. [229] dissected mouse spermatozoa and found that PNT, not the nucleus, activates the oocyte efficiently.

According to Kono et al. [230], the male pronucleus of a fertilized mouse egg can activate an unfertilized oocyte when transferred into it. The female pronucleus has less ability to do so. This could be explained by assuming that the developing male pronucleus collects more histone of sperm origin than the female pronucleus (Figure 7H). Nuclei of two-cell embryos (Figure 7J) still have oocyte-activating ability, but not those of four-cell embryos (Figure 7K). How sperm histone actives oocytes is unknown, but it may activate Toll-like receptor 9 in the oocyte’s plasma membrane as it happens in pancreatic tumor cells [231].

## 17. Polyspermy block, with a note on human diploid-triploid mosaics

In newts and birds with large oocytes, many spermatozoa, sometimes hundreds, enter each oocyte. Each spermatozoon carries such oocyte-activating proteins as protease and citrate synthase [232, 233] and multiple sperm entry is necessary for oocyte activation. Interestingly and importantly, only one of many sperm nuclei that enter the oocyte fuses with female pronucleus, while all others degenerate.

In mammals, multiple sperm entry into the oocyte is detrimental. It results in the death of the zygote/embryo. Two mechanisms exist to protect the oocyte from the danger of polyspermy: the zona reaction and the plasma membrane block to polyspermy. The zona reaction is a rapid series of chemical changes in the zona pellucida that prevents excess spermatozoa from entering/passing through the zona. It involves partial hydrolysis of the zona’s protein by proteases of CGs that are released from the oocyte’s cortex upon the entry of the first spermatozoon into the oocyte [234–237]. In the mouse, the zona reaction completes in less than 5 min after sperm contact (fusion) with the oocyte’s plasma membrane [238]. In humans, it completes in less than 10s [239].

In some animals (e.g., the rabbit, vole, and bat), there does not seem to be a functional zona reaction. Spermatozoa may keep passing through the zona even after fertilization by the first spermatozoon such that numerous spermatozoa accumulate within the PVS of a fertilized egg. In this case, it is the oocyte’s plasma membrane that blocks the entry of excess spermatozoa into the oocyte. The plasma membrane block to polyspermy occurs in oocytes of many other species. Its mechanism is not very clear. According to Bianchi et al. [199], the membrane fusion protein JUNO disappears quickly from the mouse oocyte plasma membrane after the first spermatozoon fuses with the oocyte. JUNO-less oocytes are then unable to fuse with excess spermatozoa. How JUNO proteins are released from an oocyte’s plasma membrane is unknown. How proteasomes [240] and proteases released from an oocyte’s CGs are involved in the polyspermy block of the oocyte’s plasma membrane remains to be studied further.

It is important to note that the plasma membrane of the normally fertilized egg is not completely refractory to excess spermatozoa. When naturally fertilized hamster and mouse embryos at the two-cell and four-cell stages were freed from their zonae pellucidae and then exposed to the additional spermatozoa, a considerable proportion of the early embryos were penetrated by (fused with) the additional spermatozoa. After the four-cell stage, the plasma membranes became unable to fuse with spermatozoa [183, 184, 241]. More interestingly, the plasma membranes of two- to four-cell embryos that developed after parthenogenetic activation or intracytoplasmic sperm injection (ICSI) fused more readily with additional spermatozoa than plasma membranes of embryos developing from natural fertilization [242–244]. It is possible that intermingling of sperm and oocyte plasma membranes, as clearly demonstrated by Gaunt [245], contribute to the oocyte plasma membrane block to polyspermy. At any rate, the polyspermy block at the level of the egg plasma membrane is very interesting and very puzzling [246].

**Figure 8:**
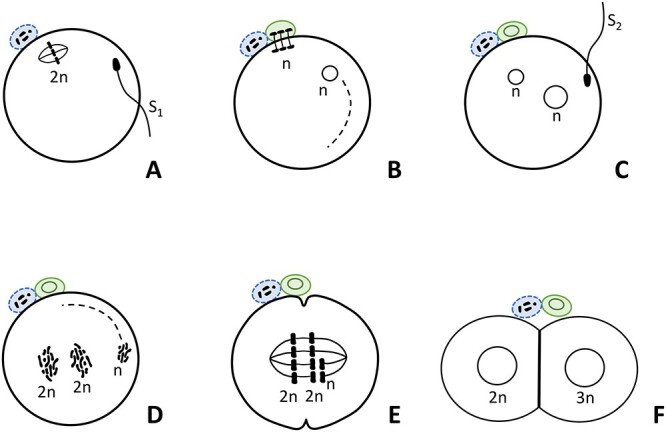
A presumptive cause of human diploid-triploid mosaicism. (A) The first spermatozoon (S_1_) enters the oocyte and stimulates resumption of the second meiotic division (2n). (B) A pronucleus is formed from S_1_ nucleus. The rest of S_1_ sperm components breaks down and a second polar body is formed. (C) The fertilized egg normally rejects the entry of excess spermatozoa, but an excess spermatozoon (S_2_) may enter the fertilized egg as shown. (D) The nucleus of the second spermatozoon decondenses while chromosomes of the S_1_ sperm and those of egg duplicate and mingle. (E) Chromosomes (n) of S_2_ spermatozoon mingle with chromosomes of one of two blastomeres of cleaving egg. (F) This results in the production of a two-cell embryo with one diploid blastomere (2n) and one triploid blastomere (3n). This embryo develops into a 2n/3n mosaic individual.

The cause of human diploid-triploid mosaicism can be either gynogenic or androgenic. As conjectured by Brems et al. [247], and experimentally established by animal (mouse) experiments [248], fusion of the second polar body with one of the cells of a two-cell embryo results in the production of a diploid-triploid individual. Another likely cause of diploid-triploid mosaics is the delayed entry of a spermatozoon into an already-fertilized egg [249]. Excess spermatozoa commonly do not enter the egg, but it could happen in some eggs, resulting in the production of two-cell embryos with one diploid and one triploid blastomeres (Figure 8). This zygote will develop into a mosaic individual. This experiment is yet to be performed.

Finally, it is important to note that the oviduct, the natural environment of fertilization, secretes various molecules that maximize the efficiency of monospermic fertilization (see Coy and Aviles [250], Coy and Yanagimachi [251], Braganca et al. [252]) and the development of preimplantation embryos. Although fertilization is possible without oviducts (e.g., by IVF and ICSI), it does not mean that the oviduct is unimportant. Most readers of this chapter and I started our lives within our mother’s oviduct as stated previously. We still have to learn much more about what is going on within the oviduct before, during, and after fertilization.

## 18. Sperm centrosome and embryo development

Centrosome and microtubules play a central role for close approximation and union of male and female pronuclei within a fertilized egg as well as the subsequent cleavage divisions of the embryos. In laboratory rodents (e.g., the mouse), centrosomes are within the oocyte, not in the spermatozoon [253]. In many other animals (e.g., monkey sheep, cattle, and rabbit), on the other hand, the fertilizing spermatozoon introduces a centrosome into the oocyte [254–256]. The sperm centrosome becomes the center of the formation of a microtubular network that brings sperm and egg pronuclei to the center of a fertilized egg [257–260]. Introduction of a defective centrosome by a spermatozoon would inevitably result in abnormal development and/or death of an embryo. Since the oocyte’s cytoplasm seems to have dormant centrosomes and microtubule-forming materials, removal of a defective centrosome from spermatozoon prior to injection into an oocyte may lead to the development and birth of a normal baby. Morita et al. [261] showed that this is likely to occur. They removed the centrosomes from rabbit sperm heads by sonication and injected the centrosome-less sperm heads into oocytes. The sperm aster was not formed, but the oocyte’s centrosome was “awakened” to fulfill the sperm centrosome’s function. Although Morita et al., followed the development of eggs up to the four-cell stage, they did not determine if the fertilized eggs could develop into fertile offspring. If a human male’s infertility is suspected to be due to a sperm centrosome problem [260], removal of the neck and tail from a spermatozoon prior to ICSI could solve the problem.

## 19. Fertile life of human oocytes and spermatozoa in oviduct

Human females are unique in that they, unlike females of most other mammals, do not have a distinct behavioral estrus, and therefore, mating (coitus) may occur any time before and after ovulation. According to Schwartz et al. [262], pregnancy after artificial insemination is best achieved when it is performed between 4 days before and 2 days after the estimated day of ovulation. This means that human spermatozoa can live up to 4 days within women’s genital tracts, while oocytes remain fertile for less than 1–2 days. Since ovulated oocytes are viable for a relatively short period of time, fertilizing spermatozoa should be in the oviduct as soon as oocytes enter it. If coitus occurs long after ovulation, oocytes are most likely “deteriorated” before meeting spermatozoa, even though sperm capacitation may take place more quickly in the female tract after ovulation than before ovulation, as shown by animal experiments [72]. A classic study by Blandau and Young [263] on guinea pig is noteworthy. Unlike common laboratory rodents, the average litter size of the guinea pig is 2–4 pups. These authors artificially inseminated 462 females between 4 and 32 h after ovulation. The first abnormal embryos were seen in females inseminated 8 h after ovulation. No normal embryo development followed insemination more than 20 h after ovulation, and no development followed insemination 32 h after ovulation. As it happens in the mouse [264], human oocytes aged in the oviduct may have misaligned meiotic chromosomes, resulting in aberrant meiosis and death or abnormal development of offspring. From the epidemiological point of view, spermatozoa should be in the oviduct before an oocyte enters the oviduct. Someday, methods simpler than currently available urine LH tests [265] could be developed to detect or predict the time of the LH surge prior to ovulation.

## 20. Effect of light on eggs and embryos

In aquatic animals such as fish, amphibians, and marine invertebrates, eggs are shed into the water and may be exposed to direct sunlight, which is rich in UV light. Their eggs have UV absorbing proteins containing mycosporine-like amino acid to protect against DNA damage [266, 267]. Ever since fertilization became “internal” during the evolution of mammals, no UV or near UV light reached the inside of the female genital tract, and therefore, eggs and embryos may have lost anti-UV (sunscreen) molecules such as mycosporine.

Today, we routinely handle mammalian spermatozoa, eggs, and preimplantation embryos under visible light before, during, and after assisted fertilization. Sometimes, they may be exposed to UV and near UV light. Daniel [268] first reported delayed cleavage of rabbit eggs by visible light. Hirao and Yanagimachi [269] found that near-UV light emitted from ordinary fluorescent lamp disrupts the second meiosis of hamster oocytes. Shielding the light with a red filter protected oocytes from the detrimental effect of light. Vulnerability of hamster oocytes to light was confirmed by other investigators [270, 271].

The effect of light is likely due to the production of reactive oxygen species within the oocyte’s cytoplasm [272, 273]. Hamster eggs seem to be exceptionally vulnerable to light. Taurine included in fertilization and embryo culture medium [75] seems to act as an antioxidant [274]. Although mouse eggs are less sensitive to light than hamster eggs [273], a detrimental effect of light on embryonic development in the mouse is a certain possibility [275]. Since it is well known that hybrid mice are more resistant to various environmental stresses than are inbred mice, negative results obtained by experiments using hybrid mice [276] should be taken with caution. Whether or not light is detrimental to embryo development of the rabbit [268, 277–279] and human [280, 281] has been controversial. Further studies are needed to clarify the effects of intense light on gametes and embryos of various animals and humans. It is possible that eggs and embryos of some women are deficient in the ability to protect against oxygen radicals generated by light. Minimizing exposure to intense light and addition of antioxidant to the medium may increase the chance of fertilization and normal embryonic development before transfer to females. Many IVF clinics are now using Embryoscope to capture thousands of images using light microscopy to determine which embryos are best to transfer. Repeated exposure of embryos to intense light should be done with caution.

## 21. Puzzles of seminal plasma and sperm competition

In 1988, O et al. [282] reported the loss of many hamster embryos after mating females with males whose accessory glands had been partly or completely removed. In these females, fertilization proceeded normally, but many embryos died during their post-implantation development. Later, it was found that seminal plasma protects spermatozoa from oxidative stress [283], which may alter the DNA methylation pattern of imprinted genes in embryos [284]. These reports need further confirmation.

In the rabbit, cattle, and humans, semen (a mixture of spermatozoa and seminal plasma) is deposited in the vagina. Spermatozoa swim out of the seminal plasma to pass through the mucus-filled cervix to enter the uterus. The seminal plasma is left behind in the vagina. In common laboratory rodents such as the rat and hamster, semen is deposited deep in the vagina, but it is quickly transported to the uterus [6] perhaps by rhythmic contractions of the cervix. Spermatozoa in the uterus then enter the oviduct through the UTJ, leaving the seminal fluid behind.

The seminal plasma is composed of secretions from various male accessory glands (ampullary glands, seminal vesicles, prostate glands, bulbourethral glands, and preputial glands). The balance of secretions from different glands seems to be important for survival of spermatozoa within the female tract. The absence or dysfunction of any of these gland secretions seem to be detrimental to spermatozoa within the female tract. According to Kawano and her colleagues [285–287], proteins secreted by the seminal vesicles of mice are important for the survival, capacitation, and fertility of spermatozoa within the female tract. It should be noted that fertilization is possible without these proteins. It is well known that high proportions of oocytes are fertilized in vivo after uterine or oviductal deposition of spermatozoa suspended in a simple defined medium without any seminal plasma components. It seems that the seminal plasma proteins deposited in the female genital tract after natural mating somehow maximize the efficiency of fertilization in vivo [288, 289]. Requirements for in vitro fertilization seem to be different from those for in vivo fertilization. Readers are referred to Bedford [290] for his opinion of the role of seminal plasma in fertilization.

When two or more males mate with a single female, what will happen? Will spermatozoa from different males compete with each other for fertilization? Many investigators maintain that sperm competition is real. The fruit fly *Drosophila* has been one of the favorite animals for the study of sperm competition. Price et al. [291] stated: “In many animals and most insects, the second male to copulate with a female typically sires most of her offspring.” How should we interpret this?

Before discussing sperm competition, I would like to mention the mating behavior of the golden (Syrian) hamster (*Mesocricetus auratus*), which I believe is very suitable for the study of sperm competition. My associates and I used this animal to study the process and mechanism of fertilization for many years and we observed their mating many times. It is important to remember that the golden hamster is a solitary animal and therefore males and females should be kept separately and individually after weaning. If they are kept in a single cage, they fight and hurt each other. Females reach full maturity in about 2 months after birth, males in about 3 months. The mature female has a very stable 4-day estrous cycle. The day of ovulation is characterized by the presence of a slightly yellowish vaginal mucus with a distinct odor, which can be squeezed out of the vagina by gentle finger pressure. Inspection should be done in the morning. The day of this vaginal mucus discharge is called Day 1 of the estrous cycle. Ovulation occurs in the early morning of this day under ordinary lighting conditions. The vaginal mucus turns to a waxy material on the next day (Day 2). The female comes into heat (behavioral estrus) on the evening of Day 4 [292]. The estrous female is characterized by the presence of a clear vaginal mucus and her displaying a “lordosis” posture in response to a male’s approach or an investigator’s finger stroking of her back. Ovulation takes place about 8 h after the onset of estrus [293]. Once Day 1 of the estrous cycle is determined, then the day of estrus (Day 4) can be predicted accurately several months ahead. Under ordinary light conditions (e.g., 14L:10D, 5 a.m. to 7 p.m. light and 7 p.m. to 5 a.m. dark), females come into estrus the evening of Day 4 Ovulation takes place about 8 h after onset of estrus [293], the early morning of Day 1. The onsets of estrus and ovulation of the hamster can be altered by changing lighting conditions at the time of weaning. Gestation of the hamster is 16 days.

When a female hamster comes into estrus (heat), she cannot be distracted. She will mate on a brightly illuminated desk and even in a bucket. The male ejaculates after repeated intromissions. Ejaculation can be distinguished from intromission by a prolonged resting interval (~20 s or so, licking his genitalia) before the male resumes mounting the female. A mating session lasts 30 min or longer. The female then goes out of “heat.” If the female is approached by the same or any other male, she bites them. It is interesting that estrus ends after the female receives sufficient semen (spermatozoa). Perhaps, oxytocin/prolactin release from the pituitary terminates the female’s estrus. If the female does not encounter a male(s), then “heat” will last 10 h or so (Yanagimachi, unpublished observations). During the preparation of this manuscript, I found reports by Lisk and Baron [294] who stated that a female hamster accepts a second male for less time (~20 min), and far less for third and fourth males (~12 and ~6 min, respectively). This contradicts my observations. Whether behavioral estrus (acceptance of male) lasts even after the female receives “enough” spermatozoa remains to be determined.

I did a series of experiments (unpublished) to see if hamster spermatozoa from two males compete for fertilization in vivo. Breeder males of proven fertility were used. Some were albino and the others wild type (brown). Females were all albinos. When an albino female was allowed to mate with two males (albino and wild-type), both tried to mount the female. Initially, males disrupted each other’s efforts, but soon they mounted the female alternately. Both males were allowed to mate for 30 min or so until the female became hostile to males (bit them) and the mating session ended. Each female delivered about the same number of albino and wild-type pups. When an albino female first mated with an albino male for 15 min, then with a wild-type male for the next 30 min or vice versa, each litter was always composed of both albino and wild-type babies. These results indicate that spermatozoa that fertilize oocytes in vivo are not necessarily the ones that enter the female tract first. In another series of experiments, albino females were mated with albino males. About 2 h after the end of copulation, females were anesthetized using methoxyflurane vapor and the uterus was exposed by laparotomy. Spermatozoa within two uterine horns were removed by flushing uteri with Ringer’s solution before spermatozoa from a wild-type male were put in each uterine horn. Sixteen days later, females delivered both wild-type (black eyed) and albino babies, indicating that fertilizing spermatozoa are not necessarily the ones that enter the uterus first.

Kenneth Y. Kaneshiro of the University of Hawaii who studied mating behavior of Hawaiian Drosophila for many years [295, 296] stated: “At least in *Drosophila*, most of the competition among males occur prior to mating. If such competition took place post-mating, i.e., within the spermatheca, then there would not have been strong selection for the evolution of such complex mating behaviors. In general, females do not mate multiple times in nature and in the laboratory. While there could be secondary mating’s under crowded conditions, these occur only when a mating is disrupted and the female is not able to receive spermatozoa to fill its spermatheca. Under natural conditions, females are very selective in mating with males that are able to satisfy their courtship requirements and males must perform an appropriate courtship display that would lower the threshold of receptivity in the female that he is courting. The male expends a lot of energy going through his courtship repertoires to be acceptable to the female, which means that courtship plays a key role in sexual relations and sexual selection in the group” (personal communication).

In my opinion based on surveying the literature and the results of my own experiments: (1) male to male competition, (2) female’s choice, and (3) male’s age and luck (e.g., chance of meeting female as well as the time after the last ejaculation), rather than sperm-to-sperm competition, determine fertilization success in vivo. This is, of course, the subject of open debate.

## 22. Similarity between sperm and neurons

**Figure 9:**
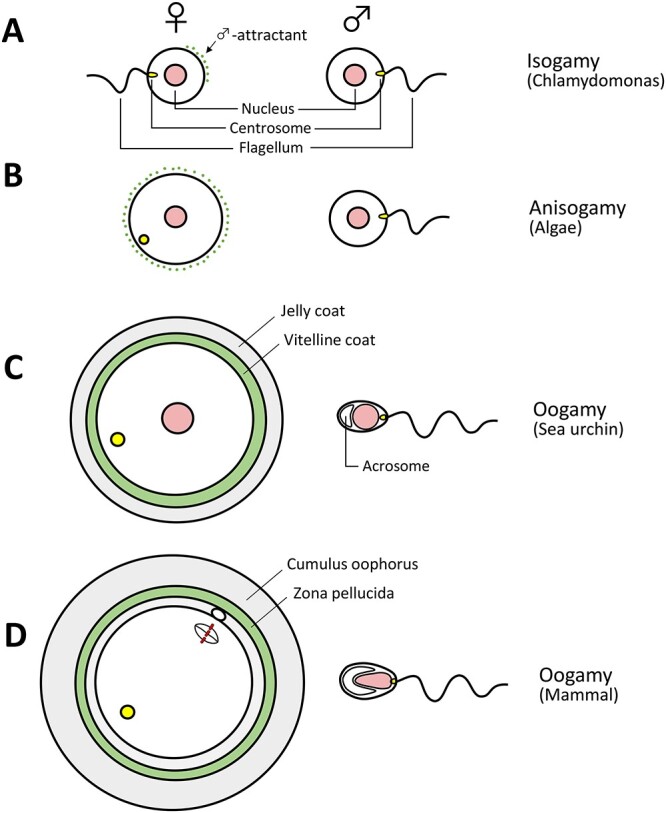
Comparison of male and female gametes of various organisms we see on the Earth today. For explanation, see the text.

After studying the mammalian sperm acrosome reaction for many years, Stanley Meizel [297] published a review entitled: “The sperm, a neuron with a tail: ‘neuronal’ receptors in mammalian sperm.” Receptors he listed included: adrenergic receptors, GABA receptor channels, nicotinic acetylcholine receptors, and olfactory receptors among many others. Meizel maintained that these receptors play essential roles in sperm acrosome reactions during normal fertilization. The presence of a variety of “sensory receptors” (including odorant receptors) in spermatozoa has been reported [298]. The presence of neuronal (sensory) receptors may not be surprising. When our ancestors were unicellular organisms, they had to reproduce by cell division. During the course of evolution, two cells united and exchanged genetic information before they multiplied further. This was the beginning of fertilization. Cells that combine genetic information from different individuals can be called gametes. Cells in the gamete stage must have had some molecules to facilitate their union. The molecules can be called “sensory (neuronal) mutually-attracting molecules.” In the early evolutionary stages of sexually reproducing organisms, all gametes must have resembled each other (Figure 9A). Today, we still see this type of gametes and their union, called isogamy, in yeasts and *Chlamydomonas*, for example. When one type of gamete has evolved to become larger than the other and is typically motionless, while the other type of gamete has become small and typically motile, their union is called anisogamy or heterogamy (Figure 9B). Today, we see this in brown algae, for example. When one type of gamete cell has evolved to become a much larger nutrient-storing motionless cell (called oocyte/egg), while the other is a small and motile cell (spermatozoon), their union is called oogamy (Figure 9C and D), which we see today in all animals.

In the sea urchin (Figure 9C), the mature, larger female gamete, the egg, has completed meiosis and has a haploid nucleus. Each egg is protected by noncellular coats (a thin vitelline coat and a thick jelly coat). Tiny haploid, free-swimming male gametes, spermatozoa, have an acrosome containing lysins to dissolve the egg’s coatings. In mammals, the egg coat is thick and elastic (Figure 9D). The oocyte has not completed meiosis and does not become haploid until after sperm entry into the oocyte cytoplasm.

When the ancestors of mammals were unicellular organisms, the gametes must have recognized each other by chemical means (“odor”). Is it still true today? Students of sea urchin fertilization think so [299, 300]. They have surmised that the egg itself secretes a diffusible chemical factor (chemoattractant) and its concentration gradient, which is highest at the egg surface, attracts spermatozoa toward the egg proper. However, we must remember that fertilization of many marine animals, including sea urchins, takes place in turbulent seawater that is constantly moving forward and backward, not like the tranquil water in a Petri dish. It must be examined carefully whether a concentration gradient of chemoattractant around each egg is maintained, even in turbulent water, and spermatozoa swim up to the gradient toward the egg.

In mammals, fertilization takes place within the female’s oviduct, which is not stationary at all, in particular during fertilization. In the mouse [27] and perhaps in other rodents and some other mammals, oviducts of females during the periovulatory period exhibit very active peristaltic movements that move the fluid within the oviduct forward and backward. There is no concrete evidence that spermatozoa in the oviduct are attracted by chemicals released from a live oocyte or from cumulus cells surrounding the oocyte. Even after extensive “washings” of cumulus-oocyte complex with a physiological salt solution, spermatozoa still are able to enter the cumulus to fertilize oocytes. The notion that a live oocyte keeps secreting a sperm attractant to direct a spermatozoon into it is also unlikely because oocytes killed by freeze-thawing under nonprotective conditions [301] or by storage in highly concentrated salt solutions [302] still allow spermatozoa to pass through the zona pellucida.

At any rate, the presence of many neuronal receptors in mammalian spermatozoa is very puzzling. Why are they there and what are they for? During evolution, the functions of neuronal receptors may have been altered. Instead of serving to the mutual attraction of male and female gametes, they may become involved in triggering the sperm’s acrosome reaction [297], hyperactivation, and even other events during spermatogenesis and epididymal maturation of spermatozoa (Meizel, personal communication).

## 23. ICSI: its short history and challenges to be considered

Uehara and Yanagimachi [303, 304] started sperm injection into oocytes out of simple scientific curiosity—just to see what would happen. Would the oocytes remain unchanged or start to participate in syngamy and embryo development? In any case, the result would be interesting to know. We then found that the heads (nuclei) of epididymal spermatozoa (even those of hardly motile testicular spermatozoa) could develop into normal looking pronuclei in eggs. This study initially drew no interest by others, except [305–307] who applied this technique to analyze the mechanisms of sperm nucleus decondensation within the egg cytoplasm. It was Iritani and Hosoi [308] and Goto et al. [309] who first obtained live offspring of the rabbit and cow after sperm injection into eggs. In 1992, after pioneering study by Lanzendorf et al. [310], Palermo et al. [311] reported the birth of human babies after injection of spermatozoa into eggs, which has been called intracytoplasmic sperm injection or ICSI. It was quickly found that ICSI could rescue various forms of human male infertility, including oligospermia, cryptozoospermia, asthenospermia, and teratospermia [312].

A major difference between ICSI and natural fertilization is that in the former, the entire body of a spermatozoon including the plasma membrane and the acrosome is injected into the oocyte cytoplasm (ooplasm). During normal fertilization, the sperm plasma membrane fuses with the oocyte plasma membrane and stays on the egg’s surface. Acrosomal contents which include powerful hydrolyzing enzymes are released from spermatozoa during the acrosome reaction and never enter the oocyte cytoplasm [25]. If a live spermatozoon is injected into an egg, it may keep swimming within the egg cytoplasm for some time until its plasma membrane disintegrates. The sperm nucleus then begins to decondense. Nuclear decondensation occurs quickly for some spermatozoa and slowly in others. Prior to human ICSI, the plasma membrane of the sperm tail is broken. This is done by sucking a spermatozoon tail first partially into an injection pipette, then “scraping” the tail against the bottom of dish. Since the plasma membrane of mature spermatozoon has no underlying cytoplasm and is unable to repair by itself, damaged sperm membrane will disintegrate progressively after injection into the oocyte cytoplasm. The speed of sperm membrane disintegration within the egg cytoplasm would vary from spermatozoon to spermatozoon. Consequently, the time when SOAF is released into the egg cytoplasm would vary from egg to egg. If the sperm plasma membrane is removed prior to ICSI, egg activation occurs much faster. This was proved to be the case [313].

We tend to speculate that normal-looking spermatozoa with good motility are genomically “normal,” whereas those with poor motility and deformed heads are genomically abnormal. Burruel et al. [314] studied this by using BABL/c mouse. This mouse strain has been used extensively for studies of cancer, immunology, and cardiovascular diseases, but it is one of least fertile strains of mice. About 70% of their spermatozoa are deformed, many of them being grossly abnormal in head structure. We were able to produce healthy offspring after injection of spermatozoa with grossly abnormal heads. Although the incidence of genomic abnormalities seems to be higher in deformed spermatozoa than in normal-looking spermatozoa, not all the spermatozoa with deformed heads are genomically abnormal [315].

Will it be possible to assess genomic status of spermatozoa without “killing” them? Methods proposed by Watanabe et al. [316] and Yang et al. [317] are very labor-intensive and time-consuming; therefore, conventional genomic analyses of cells from preimplantation embryos [318, 319] are still the best way today to avoid the birth of offspring with serious developmental problems.

The volume of the acrosomes of some species (e.g., those of the hamster and guinea pig) is very large relative to the volume of the entire body of the spermatozoon, and therefore, injection of an acrosome-intact spermatozoon of such species inevitably results in the death of the oocyte [320]. Acrosomes must be removed prior to ICSI to avoid the death of oocytes. The cause of oocyte’s death is unknown, but the cytoskeletal system seems to be extensively damaged as evidenced by the deformation of the oocyte prior to its disintegration. In species whose spermatozoa have small acrosomes, like those of the mouse and human, the removal of the acrosome prior to ICSI may not be necessary. However, eggs of some individuals could be vulnerable to damage by exogenous proteases such as acrosin. In such cases, the removal of the acrosomes from spermatozoa prior to ICSI would lead to a higher rate of successful pregnancy. Although Morozumi and I recommended the removal of acrosomes from human spermatozoa prior to ICSI [321, 322], this still has been totally neglected. Nevertheless, there must be some women whose oocytes are sensitive to exogenous proteases such as sperm acrosin.

Spermatozoa of many farm animals (e.g., cattle, sheep, and pig) have fairly large acrosomes. The removal of both the plasma membrane and acrosome from the sperm head prior to ICSI may increase fertilization success rate. As of today, ICSI in farm animals has not been very successful [323–328].

## 24. Fertilization by round spermatids and spermatocytes

Female germ cells, eggs, become fertilization competent during meiosis. Then, how about male germ cells? Do they become fertilization competent only after meiosis and transformation into round spermatids to spermatozoa? My colleagues and I found that the nuclei of mouse round spermatids that had just completed meiosis were able to produce live offspring after injection into eggs [329–331]. We produced five generations of mice by round spermatid injection (ROSI) and compared the fifth generation with the control (naturally bred) mice. We found no difference between the two in their growth, fertility, or behavior [332].

As of today, laboratory animals other than the mouse that have produced offspring through ROSI include: the rat, hamster, rabbit, and rhesus monkey (see Yanagimachi for review) [333]. The overall efficiency of ROSI in these animals is low and its reason is unknown. It seems that the egg cytoplasm suppresses expression of some of spermatid-specific genes (e.g., protamine1 and protamine2; [334]) and this correlates with the disruption of embryo development [335].

For successful ROSI, the eggs after ROSI must be fully activated. Do round spermatids have the ability to activate eggs? Yazawa et al. [336] studied species specificity of oocyte activation by round spermatids. They injected round spermatids of various species of animals into mouse oocytes and found that spermatids of either the mouse or rat were unable to activate mouse eggs, while those of the hamster, rabbit, and human could do so even though the patterns of intracellular Ca^2+^ oscillations were not quite normal. Although human round spermatids have the ability to activate human eggs, post-ROSI stimulation of oocytes (e.g., by electric current, Ca^2+^ ionophore, or phospholipase C) enhances subsequent embryo development.

Tesarik et al. [337] were the first to report the birth of human babies by ROSI, followed by Gianaroli et al. [338] and Tanaka et al. [339, 340]. The key to success of human ROSI is the correct identification of round spermatids. Human round spermatids and spermatogonia are similar in appearance and size, but they can be distinguished from each other by the presence or absence of nucleoli. The nucleus of a spermatogonium commonly has one or a few nucleoli, whereas that of a spermatid has none. Although the presence of an acrosome vesicle is a reliable indication that the cell in question is a spermatid, its absence does not mean that it is not [340, 341]. We wish there were antibodies available that specifically bind to the plasma membrane of spermatids so that we could distinguish round spermatids from all other types of cells in the testis. As of today, such antibodies are not available.

Why do round spermatid nuclei have less ability to produce live offspring than nuclei of mature spermatozoa? It is known that the nucleus of the mature spermatozoon is loaded with small and large noncoding RNAs [342, 343], which are believed to play important roles in regulating gene activities of developing embryos [344]. Although these sperm-borne RNAs are not absolutely required for embryo development in view of parthenogenetic (gynecologic) development of oocytes following extensive gene manipulations [345], it is certainly possible that sperm-born large and small RNAs enhance embryo’s survival by ensuring correct gene expression and epigenetic setup. When we performed ROSI [329, 331], the entire contents of a round spermatid were injected into the oocyte’s cytoplasm and therefore all or almost all of the spermatid RNAs, both large and small, must have been transferred to the oocyte. However, additional injection of sperm RNAs might improve embryo development following ROSI.

We were able to obtain live offspring after injection of mature oocytes with nuclei of secondary spermatocytes [330] and even primary spermatocytes [346, 347]. However, its efficacy, in particular after injection of primary spermatocyte nuclei, was far lower than that of ROSI. Premature separation of sister chromatids within the oocytes seemed to be a major problem in the case of primary spermatocyte injection. This problem may be resolved by co-injection of cohesin to maintain sister chromatids’ adhesion, but such experiments have not been done.

## 25. Sperm sexing

Ever since the role of sex chromosomes in sex determination was clarified in the beginning of the last century, numerous attempts have been made to separate X- and Y-chromosome-bearing spermatozoa. Although many different procedures have been proposed and claimed to be successful, none were very convincing except for the one that uses flow cytometry to measure and sort DNA-stained sperm on the basis of relative DNA content. This method developed by Johnson and his colleague [348] has been used commercially in cattle breeding [349] and applied successfully to other mammals (e.g., sheep, goats, rabbits, pigs, horses, deer, cats, dolphins, and primates) as well as humans [350].

Recently, Umehara et al. [351, 352] claimed that they could isolate mouse spermatozoa by either the X- or Y-chromosome. The principle of this technique is that Toll-like receptor (TLR), known to play a key role in the innate immune system, is in the plasma membrane of the sperm tail. In the presence of the potent TLR7/TLR8 activator, resiquimod, Y-carrying spermatozoa swim faster than X-carrying ones. This makes it possible to prepare sperm suspensions rich in either X- or Y- carrying spermatozoa. Artificial insemination using sex-sorted mouse spermatozoa resulted in the birth of offspring of expected sex at ~80% accuracy.

Since numerous spermatogenic cells, including round and elongating spermatids, are connected by intercellular bridges until spermatozoa are released from Sertoli cells [353], it is somewhat difficult to conceptualize how TLR is assembled in/on the plasma membrane differently in X- and Y-spermatozoa. According to Chen et al. [354, 355], X- and Y-sorted bull spermatozoa contain several different RNAs and proteins. Since the number of spermatozoa used for insemination was rather small and the X- and Y- sperm separation rate was not 100%, further validations are needed. For the current status of X- and Y- sperm separation, readers are referred to a review by Rahman and Pang [356].

In humans, sexing spermatozoa is very desirable for men who do not want to transmit their infertility to their sons due, for example, to severe Y-chromosome aberrations. Will it be possible to identify and purify viable X-bearing spermatozoa? Today, we do not need millions of spermatozoa for successful fertilization and pregnancy. Theoretically, a single good spermatozoon is all we need when the ICSI technique is used for insemination. Although X-bearing spermatozoa can be distinguished from Y-bearing ones by fluorescent in situ hybridization [357] with 100% accuracy, spermatozoa would all be “dead” by the end of diagnosis. As of today, there is no other simple, noninvasive method available for identification and isolation of viable X- and Y- spermatozoa.

## 26. Conversion of somatic cells to germ cells—artificial gametes

Some women and men have neither mature germ cells (oocytes and spermatozoa) nor their precursor cells in ovaries or testes. There are likely multiple causes for the lack of mature germ cells in these individuals. During embryogenesis, primordial germ cells may have failed to enter developing gonads. Genetic factors such as Y chromosome problems and nongenetic problems such as diseases, accidents, surgery, medications, toxins, and radiation can be the causes of the absence of spermatozoa and oocytes in the testis and ovary, respectively.

At least in the mouse, it is now possible to convert adult somatic cells to induced pluripotent stem (iPS) cells and then to mature oocytes and spermatozoa after a series of extensive gene manipulations [358–361]. To produce human spermatozoa and oocytes from iPS cells, neither animals nor their organs, tissues, or even cells should be used. Humans are emotional creatures. We must put ourselves in the position of the individual who may be born after such cell manipulations.

What we need for fertilization are haploid male and female cells with proper genomic imprinting. They need not require motile tails (for sperm) or a large amount of cytoplasm with nutrients (for eggs). Someday, it should become possible to convert adult somatic cells (e.g., skin cells, hematopoietic stem cells, or hair follicle cells) directly into haploid cells. As long as they have a haploid set of chromosomes with proper male and female genomic imprinting, they could be used as gametes. The central scientific issue is the induction of meiosis in somatic cells.

It has been known for a long time that pairing of homologous chromosomes can occur in adult somatic cells [362–364]. It has been thought that homologous chromosomes tend to attract each other and then an extended prophase, either natural or artificial, leads to the pairing of homologous chromosomes. According to Adhikari et al [365], prolonged arrest of oocytes at the prophase of the first meiotic division is due to phosphorylation inhibition by cyclin-dependent kinase 1 (CDK1). If we can arrest mitotically active cells (e.g., skin stem cells, hair follicle cells, or hematopoietic stem cells) at the prophase of mitosis for an extended period of time, they may begin meiotic divisions. When the cells are freed from the inhibitor, they may initiate meiotic divisions, culminating in the production of haploid cells. Finding and manipulating genes such as the mammalian homolog of Mei2, which controls initiation of meiosis in yeast [366, 367], may allow somatic cells to initiate meiosis. Of course, erasure and re-establishment of proper sex-specific genomic imprinting in these cells must take place simultaneously. Recently, Hirosawa-Takeda et al. [368] and Oura et al. [369] found that the zinc-finger protein (ZFP541) gene is involved in the initiation of meiosis of male germ cells. Whether activation of such genes in somatic cells induces meiosis is of academic interest.

## 27. Transfer and exchange of sperm chromosomes between two individuals

Transfer and exchange of chromosomes (genes) take place routinely by conventional animal and plant breeding. Transfer of chromosomes in the meiotic spindle of an oocyte of an individual to an enucleated oocyte of another individual has been done in the monkey [370]. Would it be possible to exchange a single chromosome (for example, Y chromosome) between two different human individuals?

Y chromosome microdeletions cause severe oligospermia in men. Although microsurgical injection of a single spermatozoon into an egg may overcome male sterility, the genetic defect would be transmitted to their sons. Repairing a defective Y chromosome or replacing it with a good Y chromosome would be the better way to solve the problem. In the case of Y chromosome donation, the Y chromosome of any fertile man should function. Since the Y chromosome does not carry genes essential for daily life, the Y chromosome of any other individuals could be used. However, the Y chromosome of a fertile man in the family on the father’s side of the recipient would be preferable. Chromosome sorting procedures that are currently available [371, 372] seem to be rather harsh. It is very unlikely that Y chromosomes thus separated can be used for therapeutic purposes. As the technique improves, however, removal of a defective Y chromosome and its replacement with a normal one could become possible.

## 28. Life without males

There are animals that reproduce just fine without males (or females, for that matter). Some sharks and lizards are examples. A Hawaiian lizard, the mourning gecko (*Lepidodactylus lugubris*), reproduces parthenogenetically without males. Interestingly, infertile males appear from time to time [373], indicating that this lizard was previously gonochoristic. Incidentally, these infertile males produce spermatozoa, but the heads and tails are all separated. It should be noted that parthenogenetic lizards maintain their genomic diversity by recombining sister chromosomes, rather than homologous chromosomes to maintain heterozygosity [374]. In birds, parthenogenetic development is mostly abortive [375]. In mammals, “parthenogenesis” is possible only after extensive manipulation of imprinted genes in eggs [345, 376, 377]. At least two eggs are needed to produce one “gynogenetic” female. Imprinted genes prevent a normal egg from undergoing parthenogenesis [378].

Most likely, life on the earth started without males. The gonochoristic (bisexual) mode of reproduction via fertilization emerged during evolution and has been maintained in most animals including mammals. Although unisexual (female only) reproduction can reproduce offspring quickly, changes in the environment (including diseases) may wipe out all individuals of the species due to the lack of genetic diversity. Almost all animal species on Earth today have bisexual modes of reproduction (union of sperm and egg), which allows the mingling of genetic information of two different individuals, male and female. Even species that reproduce asexually have methods of sharing genomes to increase genetic diversity (spores in yeast, plasmid in bacteria, etc.).

In the advent of somatic nucleus transfer technology, it is now possible to produce offspring without males. All we need are females. An old male cat, for example, can be cloned as follows: (1) collect leucocytes of the blood of this cat to isolate their nuclei, (2) collect recently ovulated oocytes from a young female cat and remove metaphase II nuclei, (3) inject a leucocyte nucleus into the enucleated oocyte, and (4) activate the now diploid egg either chemically or physically to allow it to begin embryonic development before transfer to the egg-donor cat. Theoretically, only one young “volunteer” female cat is needed to clone an old cat. In reality however, because cloning is so inefficient, more than one young cat would be needed to clone an old cat as most of manipulated oocytes and embryos would die.

Theoretically, hundreds and thousands of cloned individuals can be produced from a single male or female. Is this what we really want to do? Soon after we succeeded in mouse cloning [379], we had opportunities to discuss “cloning” with people inside and outside of the University of Hawaii. Most of our audiences were not scientists. After the question-and-answer session, I asked the audience: “Ladies, as you see now, it is possible to have the world without males. Imagine the world without men. Far less crimes and no more war. What do you think of that.” Several ladies stood up saying: “No, we do not want to live in a world without men.” “Why,” I asked? Answers were unanimous: “ our life would be boring,” “we need help from them,” “ we do not want to live in the world without men.” Yes, men, we are all needed.

It is most likely that life on the surface of the Earth began without males or females. All individuals were essentially “females.” Modified individuals then appeared to assist reproduction of females. They are “males.”

Today, there are currently 29 countries where same-sex marriage is legal. Certainly many of the couples desire to have their genetic children. Female–female couples may have genetic children as already mentioned. How about male–male couples? It may be possible to covert male A’s somatic cells to haploid cells with female genomic imprinting. Then, put the haploid nucleus (of male A) and one normal spermatozoon (of male B) in a donated mature oocyte from which the nucleus has been removed previously. This egg would develop into an offspring with genomic information from two males, A and B plus the mitochondria genome from the oocyte donor. The eggs receiving two Y chromosomes (from males A and B) would not develop into live offspring. Embryos without X chromosome would not develop to livable offspring as this chromosome carries many genes necessary for a diverse range of cellular functions. We are in the era of what we can do and what we should not do.

## 29. Human and organ cloning

Soon after we published a paper describing the first cloned mice [379], we received many telephone calls from news reporters. Their primary interest was not mouse cloning, but human cloning. “Will it be possible to clone a human now? If not, how soon?” Ever since the birth of “Dolly”—the first cloned sheep—, countless papers were published about human cloning. Human cloning is indeed a popular, yet very controversial subject. While cloning pet animals (cat and dog) met little resistance from the general public, cloning farm animals (e.g., cattle) as foods provoked a considerable controversy. “Fear of the unknown” is natural. In my opinion, cloning of farm animals should be used in a restricted manner. For example, the production of several “superior” stud animals is reasonable. However, the exclusive use of cloning technology for the rapid production of a huge number of “superior” animals would not be advisable because sudden changes in the environment (including disease) may wipe out all of the animals due to the lack of genetic diversity.

I once received a telephone call from a man who lost his only son by an accident. He and his wife desperately wanted to revive their son by cloning. I told him that I am not a medical doctor, and the current cloning technology is not ready for practical use for humans. I fully understood the couple’s desire. Perhaps, cloning is the only way to grant their wish. But should their wish be granted? This is a question not just for scientists, but for all of society to ponder.

No one is perfect. All of us want our children healthier and happier than ourselves. We see better features and abilities in our partners and wish to transmit these to our children. Sexual reproduction makes this possible. This is what gonochoristic (bisexual) reproduction all about. Cloning would preclude such betterment in our children.

There may be someone who thinks they are perfect and wants to be born again with the same genetic constitution. There is nothing wrong with their idea. There is no reason to refute their wishes. However, whether the person thus born is pleased or not is a different story. Physical and social environments their cloned “children” face would obviously be very much different from those of their “parents.” There is no guarantee that cloned children will grow in the way their “parents” and society anticipate. The happiness of a person is of prime importance, regardless of the way they are born.

Unlike reproductive cloning, therapeutic use of cloning technology has been accepted almost unanimously by the medical community and the general public. Conversion of iPS cells to a particular type of cell (e.g., epithelial, muscular, nervous, endocrine) could be done relatively easily, but whether these cells can survive and function in the environment where the cells of our interest died or malfunctioned is largely unknown. The production of tissues from iPS cells would be even more difficult because tissues are made of many different types of cells. The production of organs from iPS cells would be difficult because organs are made of many different types of tissue cells. iPS cells can be used to construct microscopic organoids (e.g., kidney organoids [380, 381], but it is very doubtful if they can develop in vitro into large transplantable organs with properly developed blood vessels.

My proposal is as follows. Today, numerous infertility clinics store thousands of human embryos (mostly in blastocyst stage) that are kept frozen for possible transplantation to their own mothers. On request from parents, embryos are defrosted and transferred to mothers. Not all of the stored embryos have this fate. On the contrary, most embryos are defrosted sooner or later without transfer into mothers and die. With the prior consent of parents of frozen embryos, the inner cell mass cells can be removed completely from a blastocyst to produce ICM cell-less blastocyst. Meanwhile, somatic cells of a patient who desire a new organ are first converted to iPS cells. They are further manipulated such that their descendant cells are able to participate in the formation of all organs but head and limbs. When these altered iPS cells are transferred to the inner cell mass-less blastocyst previously described, they would develop to a full-term “fetus” without head and limbs. All organs of this “fetus” would perfectly match to the person who provided the original cells. Organs thus transplanted to the patient would be far smaller than adult organ but would grow rapidly. The original organs that were not functioning well could be removed at a later time. It is very important to emphasize here that organs produced this way match *only* to the original cell donor, and no one else. Today, we see so many men and women hooked up to a dialysis machine endlessly (commonly 3 times a week, for 4 h each time) for the rest of their lives. If we are able to produce functional organs (e.g., kidney, heart, pancreas,…) directly from iPS cells, it is ideal, of course, but it is a very remote possibility. Even the production of an “artificial uterus” that supports development of a blastocyst to term fetus is also currently a remote possibility. Whether embryos can develop to fully developed “trunks” without heads and limbs must be determined first using various animal models even though head-less terminal fetuses of the mouse were obtained by gene manipulation [382].

## Conclusion

Although tremendous advances have been made in technologies enabling us to study mammalian fertilization, many questions remain about how gametes function, how they meet each other in the female reproductive tract, how sperm pass through the cumulus and zona pellucida to fuse with the oocyte plasma membrane, and how male and female pronuclei are formed and fuse. Current technologies are already helping us to understand these processes, yet we need more technological development in imaging, gene manipulation, identification of biological molecules, and more fully understanding the processes of fertilization to use our knowledge for the treatment of infertility and the development of better contraceptives.
